# High-resolution metabolomic biomarkers for lung cancer diagnosis and prognosis

**DOI:** 10.1038/s41598-021-91276-2

**Published:** 2021-06-03

**Authors:** Shi-ang Qi, Qian Wu, Zhenpu Chen, Wei Zhang, Yongchun Zhou, Kaining Mao, Jia Li, Yuanyuan Li, Jie Chen, Youguang Huang, Yunchao Huang

**Affiliations:** 1 Electrical and Computer Engineering, University of Alberta, Edmonton, Canada; 2 Third Affiliated Hospital of Kunming Medical University (Yunnan Cancer Hospital), Kunming, 650118 Yunnan China; 3 Shanghai Center for Bioinformation Technology and Shanghai Engineering Research Center of Pharmaceutical Translation, Shanghai Industrial Technology Institute, Shanghai, 201203 China; 4 Shanghai Fenglin Clinical Laboratory Co., Ltd, Shanghai, 200231 China

**Keywords:** Cancer, Chemical biology, Biomarkers

## Abstract

Lung cancer is the leading cause of human cancer mortality due to the lack of early diagnosis technology. The low-dose computed tomography scan (LDCT) is one of the main techniques to screen cancers. However, LDCT still has a risk of radiation exposure and it is not suitable for the general public. In this study, plasma metabolic profiles of lung cancer were performed using a comprehensive metabolomic method with different liquid chromatography methods coupled with a Q-Exactive high-resolution mass spectrometer. Metabolites with different polarities (amino acids, fatty acids, and acylcarnitines) can be detected and identified as differential metabolites of lung cancer in small volumes of plasma. Logistic regression models were further developed to identify cancer stages and types using those significant biomarkers. Using the Variable Importance in Projection (VIP) and the area under the curve (AUC) scores, we have successfully identified the top 5, 10, and 20 metabolites that can be used to differentiate lung cancer stages and types. The discrimination accuracy and AUC score can be as high as 0.829 and 0.869 using the five most significant metabolites. This study demonstrated that using 5 + metabolites (Palmitic acid, Heptadecanoic acid, 4-Oxoproline, Tridecanoic acid, Ornithine, and etc.) has the potential for early lung cancer screening. This finding is useful for transferring the diagnostic technology onto a point-of-care device for lung cancer diagnosis and prognosis.

## Introduction

Lung cancer is the leading cause of cancer-related deaths worldwide. Approximately 2.1 million lung cancer patients were diagnosed in 2018, contributing to around 11.6% of the total new cancer-diagnosed cases^1^. The overall survival rate was 64.6% for stage I lung cancer patients and 41.2% for stage II patients^2^. However, the survival rate goes down to 27% for regional stage patients and 4% for distant stage lung cancer patients^3^. Although some techniques (such as LDCT) for screening early-stage lung cancer were invented, screening costs are expensive^4^. Different lung cancer types can also lead to distinct prognosis. Lung cancer can mainly be divided into two types: non-small cell lung cancer (NSCLC) and small cell lung cancer (SCLC). NSCLC accounts for about 83% of lung cancer cases, while SCLS accounts for 13%^5^. Cell proliferation, expansion patterns, and mechanisms of SCLC and NSCLC are entirely different, which leads to completely different treatment strategies. Therefore, it is urgent to find cheap and reliable methods to detect different lung cancer stages and types.

Metabolism is an *in-vivo* activity that occurs unconsciously. It can be used to describe all biochemical reactions in organisms under the regulation of genes and proteins^6^. Metabolites, also known as intermediate metabolites, refer to small molecule compounds produced or consumed through metabolic processes^7^. Metabolomics, which can be used to search and interpret the relationship between metabolites and pathological mechanisms of diseases^8,9^, is becoming an increasingly popular tool in medicine and life sciences. It is a relatively fast and accurate technology that can reveal new knowledge about biological systems in a local or a global manner^10^. Many different types of samples can be used for cancer metabolomics research. For example, many studies have found that serum^11,12^, plasma^13,14^, saliva^15^, urine^16^, sputum^17^ and breath^18^ can be used for discovering biomarkers of cancer.

The analysis of metabolomics approaches can be divided into targeted and non-targeted metabolomics. Targeted metabolomics is to detect specific metabolites, which can achieve absolute quantification of metabolites of interest. Targeted metabolomics approaches require a priori knowledge of target metabolites^19^. It is usually applied to validate pre-determined biomarkers identified using a cohort of non-targeted metabolic analysis^20^. Non-targeted metabolomics, on the other hand, unbiasedly detects all detectable metabolite molecules in the samples^10,21^. It can only achieve relatively quantitative measurement, but it can provide a higher resolution than targeted metabolism^22^.

Although the research on lung cancer biomarkers has advanced significantly in recent years, most studies use multivariate statistical analysis to analyze targeted metabolites. In this article, we analyze all metabolites from plasma using non-targeted metabolomics to search the biomarkers related to lung cancer and predict patient prognosis based on the lung cancer type. Different ultra-performance liquid chromatography methods, including hydrophilic interaction liquid chromatography (HILIC) and reversed-phase liquid chromatography (RPLC), and a Q-Exactive high-resolution mass spectrometer were used in the metabolomic studies. Our experiment employed multivariate statistical analysis together with machine learning classifiers to investigate and validate the biomarkers associated with lung cancer stages and types. The biomarkers and estimators discovered from the plasma sample in this study can serve as a tool for screening the early-stage lung cancer and prognosing lung cancer types. Further targeted metabolomics analysis could be used to validate our conclusion.

## Materials and methods

### Materials

Methanol, acetonitrile, and isopropanol (LC–MS grade, Optima) were purchased from Fisher Scientific (Fair Lawn, NJ, USA). Water was purified with a Milli-Q purification system from Merck Millipore (M.A., USA). Formic acid (M.S. grade) was purchased from Fluka (Munich, Germany). Ammonium formate (HPLC grade, CNW) and ammonium hydroxide solution (25% NH_3_, HPLC grade, CNW), and nonadecanoic-d37 acid (C/D/N isotopes) were obtained from ANPEL (Shanghai, China). l-2-chlorophenylalanine was purchased from Intechem Tech (Shanghai, China). Hexanoyl-l-carnitine-(N-methyl-d3) was acquired from Superlco (Darmstadt, Germany). Lyso-phosphatidylcholine (12:0) was acquired from Avanti Polar Lipids (Birmingham, AL, USA). VACUETTE blood collection tube (Greiner Bio-One) and frozen pipe (KIRGEN) were used in sample collection and storage.

### Ethics approvals

The collection of samples is conducted under the informed consent of the participants and the approval of the Ethics Committee of the Third Affiliated Hospital of Kunming Medical University (Yunnan Cancer Hospital) (Ethics File #: QT201908). All methods were performed in accordance with the relevant guidelines and regulations.

### Study design

In this study, we performed the metabolomic analysis of plasma samples using high-resolution liquid chromatography-mass spectrometry (LC–MS). Blood samples of 98 patients with biopsy-proven and biopsy-graded lung cancer and 75 healthy controls from the same age and gender-matched cohort were acquired. The *p*-value of student *t*-test for age is 0.343, and *p* value of χ^2^ for gender is 0.709. The lung cancer cohort included 48 stage-I, 7 stage-II, 26 stage-III, 15 stage IV and 2 unknown stage samples (refer to Table 1). For lung cancer type, the lung cancer cohort includes 70 samples of adenocarcinoma (ADC), 14 samples of squamous cell carcinoma (SCC), 7 samples of small cell lung cancer (SCLC), 3 samples of other types, and 4 samples of unknown type. We divide two-thirds of the samples as the discovery/training set, and the remaining one-third of the samples as the validation set as summarized in Table 1.

**Table 1:** Summary of grouping of samples.

Group	Number of Samples	Age		Histology					Gender	
		Range	Median	ADC	SCC	SCLC	Others	Unknown	Male	Female
**Discovery set**										
Stage I	30	37–65	51.5	27	2	0	1	0	11	19
Stage II	5	41–55	45	4	0	1	0	0	3	2
Stage III	17	38–70	52	8	5	1	2	1	14	3
Stage IV	12	32–65	55	10	1	0	0	1	6	6
Healthy Control	50	33–69	51	N/A	N/A	N/A	N/A	N/A	25	25
Total	114	32–70	50.5	49	8	2	3	2	59	55
**Validation set**										
Stage I	15	40–60	50	14	0	0	0	1	6	9
Stage II	2	40–50	45	1	1	0	0	0	1	1
Stage III	9	37–67	56	2	4	3	0	0	7	2
Stage IV	6	46–68	47	4	0	1	0	1	2	4
Unknown	2	57–59	58	0	1	1	0	0	1	1
Healthy Control	25	31–68	50	N/A	N/A	N/A	N/A	N/A	11	14
Total	59	32–70	50	21	6	5	0	2	28	31

### Recruitment criteria

Lung cancer plasma samples were obtained from patients with lung cancer diagnosed pathologically in the Third Affiliated Hospital of Kunming Medical University (Yunnan Cancer Hospital). The collection of all samples lasted from 2018 to 2019. Patients selected for the lung cancer group need to meet the following requirement: (1) Patients need to be newly diagnosed and have not undergone any treatment and surgery; (2) Patients were pathologically diagnosed with lung cancer. At the same time, 75 healthy volunteers confirmed by the physical examination at the Third Affiliated Hospital of Kunming Medical University were recruited as the control group. The age and sex of the control group did not have statistically different from the patient group. Volunteers in the healthy control group need to be free of major diseases (cancer, diabetes, cardiovascular disease, etc.). When taking blood samples, patients need to fast before surgery and breakfast, while the control group volunteers need to fast before breakfast. After EDTA-2k anticoagulation and 2000 g for 10 min, the plasma was centrifuged at 4 °C and stored in a low-temperature refrigerator at − 80 °C for testing.

### Sample preparation

Plasma samples were prepared using the following protocol. 400 μL solvent of methanol/acetonitrile (1: 1, v/v, containing internal standard 2-chloro-l-phenylalanine, prechilled to − 20 °C) was inserted into 100 μL plasma. The sample was then vortexed for 30 s followed by incubating at − 20 °C. After 2 h, the mixture was vortexed again and centrifuged at 12,000 r/min at 4 °C for 15 min. The supernatant (400 μL) was collected and divided into two aliquots of 200 μL, and then dried under vacuum. For the HILIC analyses, one aliquot of each sample was reconstituted in 100 μL acetonitrile/water (1:1, v/v). For the RPLC analyses, the other aliquot was reconstituted in 100 μL methanol/water (4:1, v/v). The periodically injected quality control (Q.C.) samples were prepared by mixing equal volumes (10 μL) of each plasma sample to be a pooled plasma sample. The Q.C. samples were prepared following the same protocol and periodically added for every 10 test samples throughout the analytical run.

### Metabolomic analysis and data pre-processing

We follow the similar methods by Zhu *et. al* in conducting LC–MS and LC–MS/MS as well as data pre-processing^23^. Detailed description of the parameters is included in the supplementary material section.

### Statistical analysis

The computational and statistical analysis routine^24^ was followed in this study. A total of 98 lung cancer patients and 75 healthy control participants were included in the multivariate analysis. The observed clinical characteristics of the study population are summarized in Table 1. Samples were stratified and divided into a discovery set and a separate validation set according to the lung cancer stage. For this multivariate analysis study, two-third of the total subjects were randomly chosen to compose the discovery set. We adopted the area under the curve (AUC) for each metabolic feature to separately estimate the significance. Logistic regression models with Lasso regularizations were then used to develop preliminary discriminant models to classify whether individuals have lung cancer or not (binary classification) using selected metabolites. Once the model was fitted, we used the remaining one-third of subjects as the validation set to validate the corresponding biomarkers and classification model. The performance matrices are estimated using accuracy, precision, recall, the receiver operating characteristic (ROC) curve, and area under the curve with error bars of 95% confidence intervals. For identifying the lung cancer stages (multi-class classification), similar routines were employed. Considering the imbalance of lung cancer types in the collected samples, a cross-validation cohort with an internal validation exemption method was used to determine the critical metabolites of lung cancer types. All the feature selection and model fitting methods presented in this paper use custom scripts in Python.

## Results

### Statistical data processing

Sample information about lung cancer patients and volunteer healthy control participants in each group are summarized in Table 1. There are more stage-I lung cancer cases than all other lung cancer stages, and ADC is the dominant lung cancer type. Among all the patient samples, 48% of them were stage I lung cancer, 6% were stage II, 27% were stage III and 19% were stage IV. From the histological biopsy analysis results, 75% of the patient samples were ADC, 15% were SCC, 7% were SCLC and 3% were other types (large-cell carcinoma, mucoepidermoid carcinoma, and neuroendocrine carcinoma).

A total of 9822 peaks were detected using the HILIC mode, whereas 7200 peaks were obtained using the RPLC-ESI + mode and 5501 peaks obtained using the RPLC-ESI- mode. The peaks detected out of the retention time range were removed. The peaks generated by the internal standard were also removed. There were 9811 peaks left for the HILIC mode (expressed as the HLIC data set), whereas 7039 peaks were left for the RPLC-ESI + mode (expressed as the RP Pos data set) and 5485 peaks left for the RPLC-ESI- mode (expressed as the RP Neg data set). These data sets were normalized and then used for further statistical analysis and machine learning studies.

### Multivariate modelling: healthy controls vs. lung cancer at all stages

After data normalization by the method mentioned above, the principal component analysis (PCA) was adapted to investigate the dataset, which showed a trend of inter-group separation on the score plot (Supplementary Fig. S1a, b and c). The periodically Q.C. injections were clustered in a small space in score plots, indicating the acceptable stability and repeatability of the system. Scores plots of orthogonal projection on latent structure discriminant analysis (OPLS-DA) were further constructed with the datasets, which showed a clear separation between the healthy control and the patient group (Supplementary Fig. S2a, b and c), with acceptable modeling and predictive abilities (R2X = 0.399, R2Y = 0.926, Q2cum = 0.673 for the RP Neg mode, R2X = 0.282, R2Y = 0.96, Q2cum = 0.703 for the RP Pos mode, and R2X = 0.465, R2Y = 0.962, Q2cum = 0.82 for the HILIC mode, respectively). The validation of the model was performed by the default seven-round cross-validation method and a 200 times permutation test in the OPLS-DA model (Supplementary Fig. S2d, e and f.). The results indicate that the models are valid without overfitting (R2 = 0.723, Q2 =  − 0.614 for the RP Neg mode, R2 = 0.818, Q2 =  − 0.549 for the RP Pos mode, and R2 = 0.795, Q2 =  − 0.634 for the HILIC mode, respectively). All three datasets (R.P. Neg, R.P. Pos, and HILIC) were combined into one concatenate dataset for OPLS-DA analysis to show the separation ability.

Differential metabolites were selected with the criteria of VIP > 1 using OPLS-DA and *p*-value < 0.05, and were identified according to the search results of the databases mentioned in the Supplemental Materials. The results of the differential metabolites were listed in Supplementary Table S1. The metabolites with mzCloud value (or mzVault value) and the Human Metabolome Database (HMDB) I.D.s (Supplementary Table S2) were included in the pathway analysis to investigate the possible metabolic pathways significantly affected between these two groups (Supplementary Fig. S3)^25^. Seven metabolic pathways were found to be significantly altered (*p* < 0.05). Enrichment analyses based on Kyoto Encyclopedia of Genes and Genomes (KEGG)^26^ and Small Molecule Pathway Database (SMPDB)^27^ are shown in Supplementary Figs. S4 and S5, respectively.

The AUC score was calculated for each metabolite. After sorting the metabolites with the AUC score, the best twenty metabolites were selected and deployed in subsequent classification tasks. We build three logistic regression models with lasso regularization using the most significant five, ten, and twenty metabolites. The best-five-corresponding metabolites are described in Table 2 for discriminating healthy controls and lung cancer patients. In the descending order of AUC values, the five most essential metabolites used as biomarkers are Palmitic acid, Heptadecanoic acid, 4-Oxoproline, Tridecanoic acid, and Ornithine. Two different statistical hypothesis tests (*t*-test and Mann–Whitney *U* test) were performed on the distribution of these metabolites in the two populations (healthy vs. diseased). This study used the Mann–Whitney *U* test as a non-parametric alternative to the *t*-test (*t*-test assumes that the variable follows normal distribution while the Mann–Whitney *U* test does not have that assumption)^28^. Box plots with whiskers were generated for these five metabolites to reveal their distribution in healthy people and lung cancer patients. Hypothesis testing results and box plots both reveal that these five metabolites are directly and significantly different between the two groups.

**Table 2:** Five most significant metabolomic biomarkers for lung cancer screening.

	HMDB or MetPA* Number	AUC	*P*-value (*t*-test)	*P*-value (Mann–Whitney *U* test)
Palmitic acid	HMDB0000220	0.86	5.94E−14	7.81E−16
Heptadecanoic acid	HMDB0002259	0.84	5.28E−12	1.90E−14
4-Oxoproline	METPA0228	0.83	2.65E−10	2.69E−11
Tridecanoic acid	HMDB0000910	0.81	6.95E−13	1.48E−12
Ornithine	HMDB0000214	0.81	4.60E−10	9.74E−12

*MetPA (Metabolomic Pathway Analysis).

**Figure 1:**
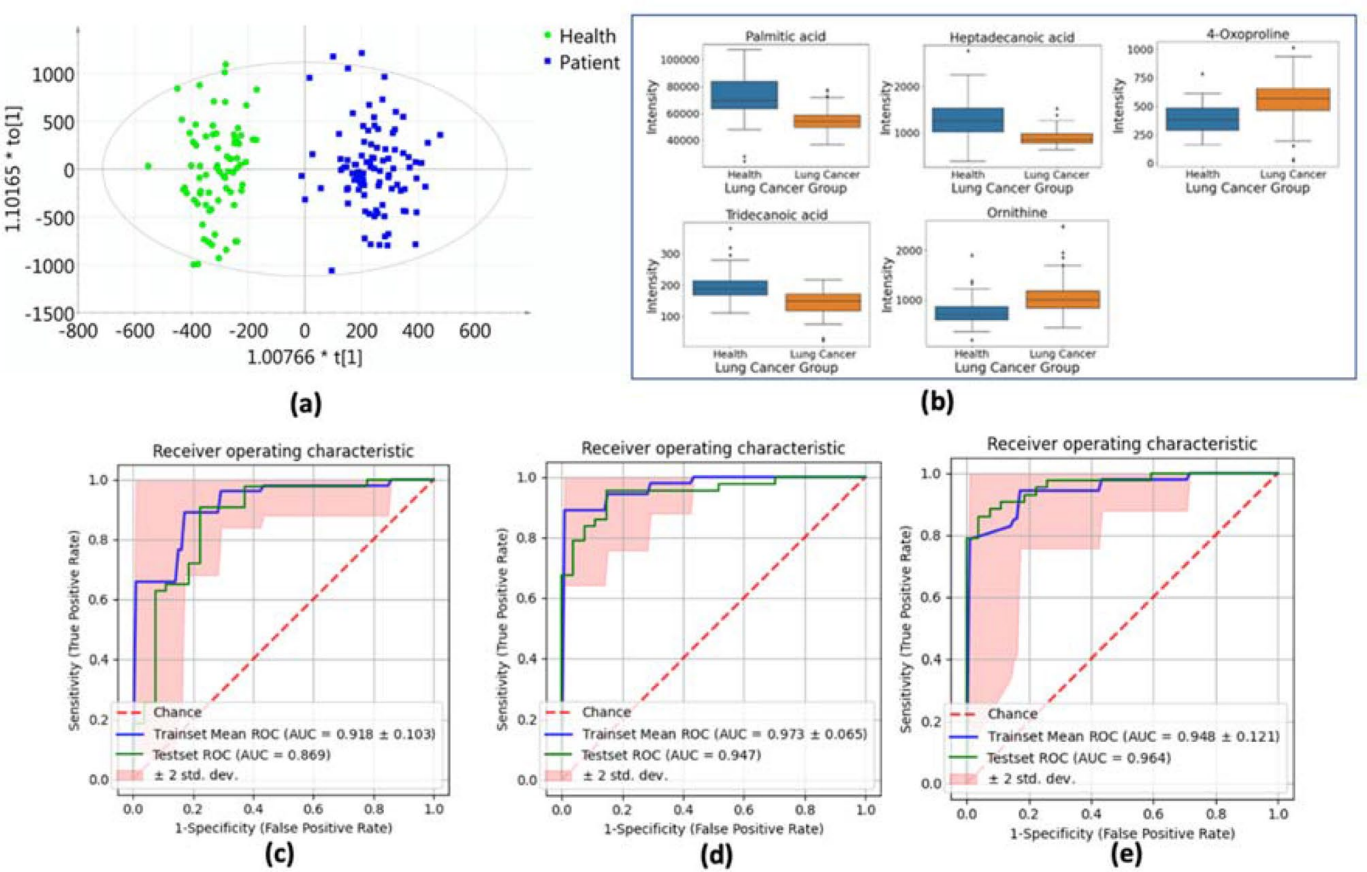
**(a)** Orthogonal projection on latent structure discriminant analysis (OPLS-DA) score plot shows the ability to separate lung cancer patients from healthy controls. **(b)** Box and whisker plots for the top 5 most important metabolites between healthy control and lung cancer groups. **(c)-(e)** Receiver-operating characteristic (ROC) curves for discriminating healthy controls and lung cancer patients [**(c)** ROC curves of the logistic model using top 5 metabolomic markers; **(d)** ROC curves of the logistic model using top 10 metabolomic markers; **(e)** ROC curves of the logistic model using top 20 metabolomic markers].

The results illustrated by the OPLS-DA score plot (Fig. 1a) and box plots (Fig. 1b) suggest that it should be sufficient to discriminate lung cancer patients from healthy people only using these candidate biomarkers. The score plot demonstrate a clear distinction between two clusters. In the box plots, the solid box represents the interquartile range (IQR), which is the distance between the 25^th^ percentile and the 75^th^ percentile of all the data points. The solid line inside the box represents the median (the 50^th^ percentile). The whiskers represent the maximum and minimum data points, excluding any outliers. Black diamonds outside the whiskers represent the outliers.

Multivariate logistic regression analysis with internal sevenfold cross-validation was performed within the discovery set. Based on the descending order of the AUC sorting score, we selected the best 5, 10, and 20 most influential metabolites as the covariates. The evaluation metrics contain accuracy, precision, recall, and area under the ROC curve. The mean cross-validation results of these logistic regression analyses of the discovery set using candidate biomarkers were summarized in Fig. 1c–e and Table 2. The blue curve denotes the mean ROC curve among the seven-folds. The reddish area represents the 95% confidence interval (CI) of the mean ROC curve. The robustness of the discovery model has been verified using the error bars (95% CI) obtained from the sevenfold cross-validation. Once the models are well trained on the discovery set, their performance can be further verified on the validation set. The results of the validation set demonstrate that the AUC score of the metabolite-only classifiers used to distinguish lung cancer patients from healthy controls is almost all above 0.83. If more minor metabolites are included, the performance metrics indicate higher accuracy, precision, and recall scores compared to using five metabolites as covariates (Table 3). The results of the independent verification team adequately proved that the classifier using only a few metabolites as the observations performed well.

**Table 3:** Performance of logistic regression models with various biomarkers for discriminating healthy controls and lung cancer patients.

	Top 5 significant metabolites		Top 10 significant metabolites		Top 20 significant metabolites	
	Discovery	Validation	Discovery	Validation	Discovery	Validation
AUC	0.918 (± 0.103)	0.869	0.973 (± 0.065)	0.947	0.947 (± 0.125)	0.964
Accuracy	0.836 (± 0.155)	0.829	0.902 (± 0.162)	0.857	0.893 (± 0.159)	0.900
Precision	0.850 (± 0.174)	0.829	0.933 (± 0.214)	0.866	0.903 (± 0.204)	0.905
Recall	0.855 (± 0.208)	0.829	0.890 (± 0.161)	0.857	0.908 (± 0.117)	0.900

### Multivariate modeling: healthy control vs. stage I and II lung cancer vs. Stage III and IV lung cancer

In the previous section, we have proved that binary classification has sufficient accuracy and satisfying robustness to separate cancer patients from healthy volunteers. However, for cancer stage screening, the binary classification may not have sufficient discriminating ability, and multi-classification tasks are required.

Lung cancer stages are classified by the TNM system, where T stands for tumour, N denotes nodes, and M stands for metastasis^29^. Each letter in the TNM system is followed by a number (and maybe also a letter) to show how advanced cancer developed^29^. In this study, we use the size of the tumour ranges from T1 to T4 to represent the lung cancer stage. Furthermore, we classify stage I and stage II patients as early-stage lung cancer (35 cases in the discovery set and 17 cases in the validation set), and stage III and stage IV lung cancer patients as advanced-stage (29 cases in the discovery set and 15 cases in the validation set), thus evolving this problem into a three-class discrimination problem.

**Figure 2:**
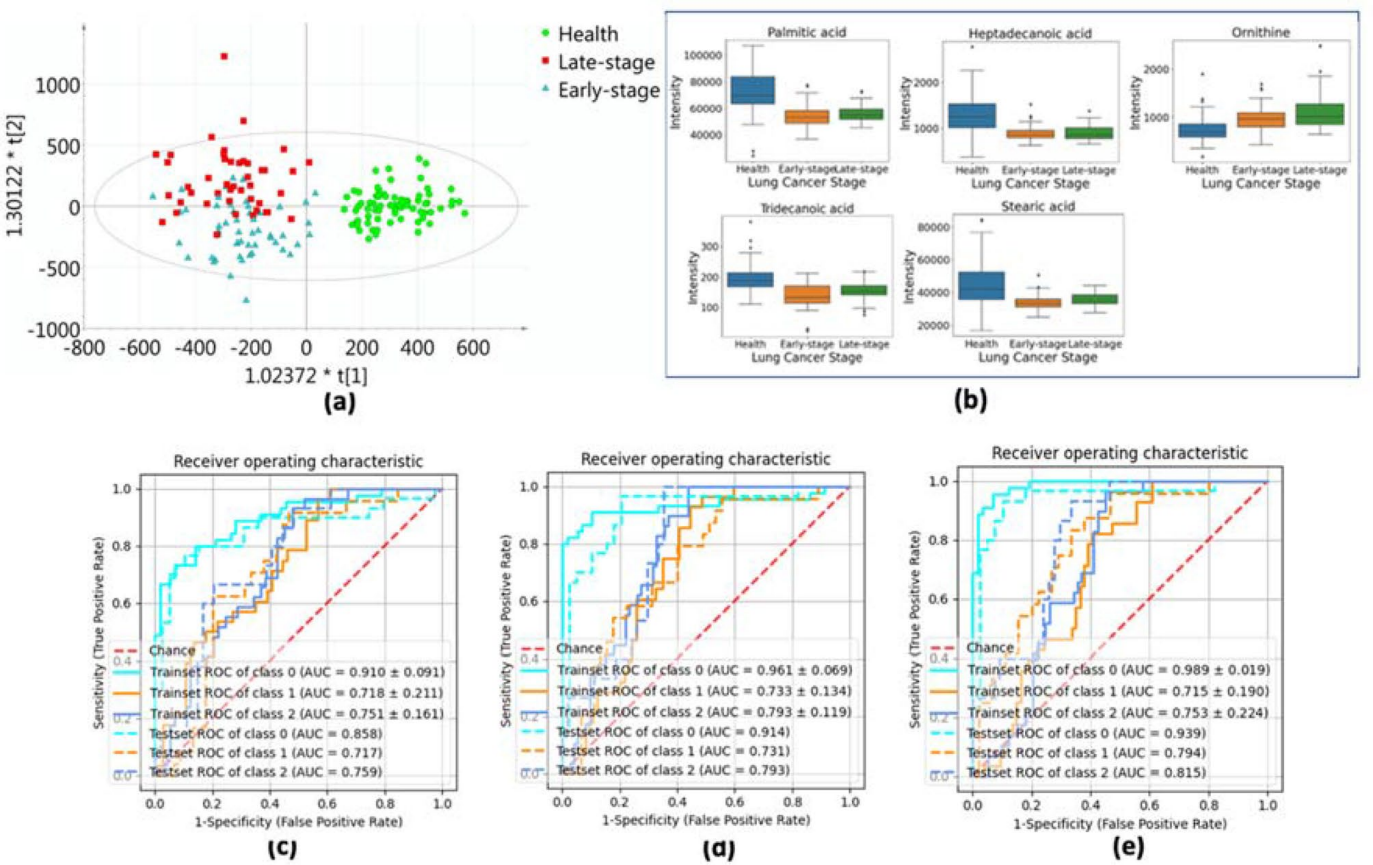
**(a)** Orthogonal projection on latent structure discriminant analysis (OPLS-DA) scores plot shows the ability to discriminate early-stage lung cancer patients, advanced-stage lung cancer patients and healthy controls. **(b)** Box and whisker plots for the top 5 most important metabolites between healthy control and different lung cancer stage groups. **(c)-(e)** Receiver-operating characteristic (ROC) curves for discriminating healthy controls, early-stage patients, and advanced-stage lung cancer patients, where class 0 represents healthy controls, class 1 represents early-stage patients, and class 2 represents advanced-stage patients [**(c)** ROC curves of the logistic model using top 5 metabolomic markers; **(d)** ROC curves of the logistic model using top 10 metabolomic markers; **(e)** ROC curves of the logistic model using top 20 metabolomic markers].

In the above section, we have analyzed the data and selected some metabolites as biomarkers. Nonetheless, biomarkers or estimators that can effectively distinguish early-stage lung cancer or advanced-stage lung cancer from healthy populations have broader applications. PCA, PLS-DA, and OPLS-DA were applied to standardized metabolomics data sets for classification. Within the two-dimensional score plot that combines both the RPLC assay and the HILIC assay (Fig. 2a), we observe that the clustering of the healthy group can be clearly separated from all the samples. The sample distribution of early-stage cancer and advanced-stage cancer has a small intersection (most overlaps occur if the early-stage samples are gathered in quadrant III while the advanced-stage group is collected in quadrant II). This means that the OPLS-DA model can efficiently distinguish whether a sample has lung cancer, but it is not particularly sensitive to lung cancer staging.

A similar routine for screening metabolites as described in “Multivariate modeling: healthy control vs. stage I and II lung cancer vs. Stage III and IV lung cancer” section is followed here. Essential metabolites are selected according to the AUC. The best-five-corresponding metabolites and its AUC scores and *p*-values are described in Table 4. For the multi-class discrimination, we use the macro average to calculate the AUC scores. In the descending order of AUC values, the five most significant metabolites that can be considered as biomarkers are Palmitic acid, Heptadecanoic acid, Ornithine, Tridecanoic acid, and Stearic acid. We built three logistic regression models with Lasso regularization with the best five, ten, and twenty metabolites. Given the distribution of these metabolites in the three groups of people, two different statistical hypothesis tests (one-way analysis of variance or ANOVA test, and Kruskal–Wallis *H* test) were performed on the three populations. Similarly, like the relationship between the *t*-test and the Mann–Whitney *U* test, the Kruskal Wallis test is a non-parametric alternative to the ANOVA test^30^. The one-way ANOVA test is considered an extension of the t-test because both assume that the variables follow a normal distribution. The Kruskal Wallis test is regarded as an extension of the Mann–Whitney *U* test, and neither assumes that the variables come from any distribution. Box plots from Fig. 2b with whiskers were generated for these five metabolites to reveal their distribution in three groups (healthy, early-stage, advanced-stage). It can be seen from the box plot that all five metabolites can distinguish healthy patients without lung cancer. Their distribution is roughly the same in the early-stage lung cancer group and the advanced-stage lung cancer group.

**Table 4:** Most significant biomarkers for discriminating healthy controls, early-stage patients, and advanced-stage lung cancer patients.

	HMDB Number	AUC	P-value (ANOVA test)	P-value (Kruskal–Wallis test)
Palmitic acid	HMDB0000220	0.77	8.08E−16	1.99E−14
Heptadecanoic acid	HMDB0002259	0.75	5.02E−14	6.56E−13
Ornithine	HMDB0000214	0.73	2.80E−09	1.71E−10
Tridecanoic acid	HMDB0000910	0.73	4.08E−13	7.32E−12
Stearic acid	HMDB0000827	0.72	5.01E−11	2.51E−10

A multiple logistic regression analysis with internal sevenfold cross-validation was performed in the discovery set. According to the descending order of AUC ranking scores, we select the best 5, 10 and 20 most influential metabolites as covariates. Figure 2c–e and Table 4 summarize the average cross-validation results of these logistic regression analyses of the discovery set using candidate biomarkers. The results of the validation set show that using only five metabolite classifiers, the healthy group, the early-stage cancer group, and the advanced-stage cancer group can be distinguished well (AUC areas are all greater than 0.7). If more secondary metabolites are included, the performance indicators show that compared with the classifier using five metabolites as covariates, the AUC area, accuracy, precision, and recall have improved to varying degrees (Supplementary Table S3). Although the effect of using one of these metabolites alone to distinguish cancer stages is not satisfactory, the results of the independent verification team fully demonstrated that the results of using more than five metabolites to train the classifier are gratifying.

### Multivariate modeling: healthy control vs. adenocarcinomas vs. squamous-cell carcinoma vs. small-cell carcinoma

**Figure 3:**
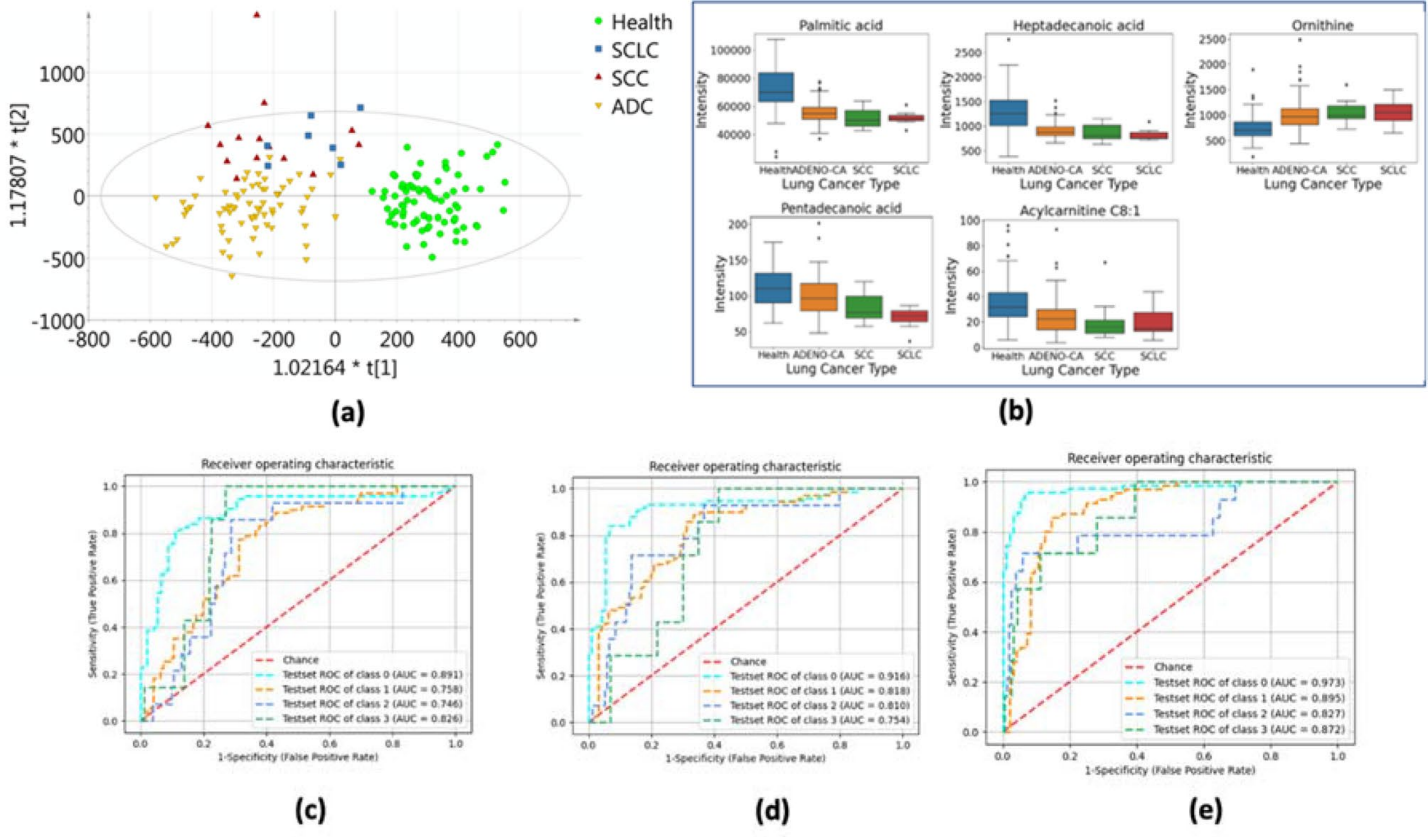
**(a)** Orthogonal projection on latent structure discriminant analysis (OPLS-DA) scores plot shows the ability to discriminate three lung cancer types and healthy controls. **(b)** Box and whisker plots for the top 5 most important metabolites between healthy control and different lung cancer types. **(c)-(e)** Receiver-operating characteristic (ROC) curves for discriminating lung cancer types, where class 0 represents healthy controls, class 1 represents adenocarcinomas, class 2 represents SCC lung cancer, and class 3 represents SCLC **[(c)** ROC curves of the logistic model using top 5 metabolomic markers; **(d)** ROC curves of the logistic model using top 10 metabolomic markers; **(e)** ROC curves of the logistic model using top 20 metabolomic markers].

PCA, PLS-DA, and OPLS-DA were applied to standardized metabolomics data sets for classification. Similar to the previous score plots, the clustering of the healthy group can be clearly separated from the patients’ group. The sample distribution of ADC lung cancer and the other two types has a small intersection. This means that the OPLS-DA model can efficiently distinguish ADC among all types of lung cancer. In the above section, we have selected some metabolites as biomarkers for binary and multi-class classification. Here we also present the best-five-corresponding metabolites for distinguishing lung cancer types, as shown in Table 5. The results showing in Fig. 3 indicate that after adding the significant metabolites to 20, our macro-AUC score can increase to around 0.89 and get an accuracy of 0.83.

**Table 5:** Most significant biomarkers for discriminating different lung cancer types.

	HMDB IDS	AUC	P value (ANOVA test)	P value (Kruskal–Wallis test)
Palmitic acid	HMDB0000220	0.78	3.99E−15	5.62e−14
Heptadecanoic acid	HMDB0002259	0.75	8.86E−13	3.75e−12
Ornithine	HMDB0000214	0.73	1.22E−07	8.76e−10
Pentadecanoic acid	HMDB0000826	0.69	1.68E−05	1.04e−05
Acylcarnitine C8:1	NA	0.69	1.67E−04	8.81e−06

## Discussions

In the past decade, an enormous amount of metabolomics studies has been devoted to using plasma, serum, or urine to explore reliable biomarkers for screening and diagnosing lung cancer. Sputum, saliva, bronchial lavage or aspirate, exhaled breath, or airway epithelium can also be collected as potential sample sources of omics-based biomarkers^4,31,32^. Most of the studies did not divide an independent adherence test cohort from all their samples to verify their findings^33,34^. Similarly, some biomarkers are identified under analyzing dozens of samples, requiring an adequate age-and-gender-matched test set to validate their conclusion^35^. In this article, we used plasma as experimental samples, and applied machine learning algorithms to screen the potential metabolite biomarkers for the lung cancer stage and type detection.

Three groups of metabolites were selected as the most significant biomarkers in our studies. For discriminating lung cancer patients, Palmitic acid, Heptadecanoic acid, 4-Oxoproline, Tridecanoic acid, and Ornithine were identified as the most significant metabolites. Palmitic acid, Heptadecanoic acid, Ornithine, Tridecanoic acid, and Stearic acid were identified as the most effective metabolites for lung cancer stages discrimination. In addition, Palmitic acid, Heptadecanoic acid, Ornithine, Pentadecanoic acid, and Acylcarnitine C8:1 were determined as the most effective metabolites for lung cancer types. The full list of the selected metabolites can be found in Supplementary Table S5.

### The impact of fatty acids

Based on our current results, many fatty acids were selected as significant potential biomarkers of lung cancer, and most of their level decrease in cancer groups (Supplementary Table S1). Cancer cells have complicated metabolic characteristics, including the Warburg effect, aberrant amino acid metabolism, and abnormal lipid metabolism, which indicate that cancer cells need high energy, high biomass (i.e., amino acids and membrane lipids) for cell proliferation^36^. Decreases in saturated fatty acids (SFAs) and monounsaturated fatty acids (MUFAs), such as palmitic acid, stearic acid, oleic acid, linoleic acid, and palmitoleic acid, have been observed in the plasma of lung cancer patients^37^, The plasma levels of SFAs and MFAs also significantly decreased in cancer groups in our study, such as caprylic acid, capric acid, undecylenic acid, undecanoic acid, dodecanoic acid, tridecylic acid, myristic acid, pentadecanoic acid, palmitic acid, heptadecanoic acid, oleic acid, stearic acid, nonadecanoic acid, and arachidic acid. The plasma levels of acylcarnitines in patients also decreased compared to the healthy controls with few exceptions. This result is consistent with our prior urinary metabolomic study of lung cancer^38^. Fatty acid synthase (FASN), a key enzyme in fatty acid synthesis, is overexpressed in most human carcinomas, including lung cancer^39^. Carnitine palmityl transferase 1C is also observed overexpressed in human lung tumors. Cancer cells show increased fatty acid oxidation and ATP production with constitutively expressing carnitine palmityl transferase 1C^40^. Levels of some lysophosphatidyecholines (LysoPC) and lysophosphatidylethanolamines (LysoPE), which are membrane lipids with proinflammatory functions, are also upregulated in our study in the plasma of lung cancer patients. Our results in this study are consistent with prior studies, including lower concentrations of fatty acids and acylcarnitines, and increased levels of lysoPC and lysoPE in lung cancer groups^37,41^.

### The impact of amino acids

Of all these identified differential metabolites, amino acids are a group of biomarkers that play a vital role in metabolism. Research on the change of amino acid concentration level in plasma and serum as important markers has been studied. Our experimental results also recognized some amino acids as the most significant biomarkers, for example, Ornithine, 4-Oxoproline, 4-Hydroxyproline, N(6)-Methyllysine, l-Cystine, l-Arginine, and N6-Acetyl-l-lysine. Proenza et al*.* compared the levels of amino acids in 14 lung cancer patients and 32 healthy controls’ blood samples and reported an increased Ornithine level in the lung cancer group^42^. The study of Cascino et al*.* with 41 lung cancer patients and 28 healthy controls also demonstrated a similar level increase in the lung cancer patient group^43^. A recent study conducted by Ni et al*.* developed a serum amino acid and acylcarnitines-based classifier to diagnose lung cancer^44^. Their targeted metabolomics method measured 13 types of amino acids for 57 lung cancer patients and 130 healthy control patients. They chose to use Arginine as one of the six important metabolites for the final classifier.

Maeda et al*.* demonstrated the development of an all-stage, multiple-type lung cancer detection test based on accurate measurements of 21 plasma amino acid concentrations^44^. The study was developed using 4340 healthy control samples and 318 lung cancer patient samples with all four stages and three types (Adenocarcinoma, SCC, and SCLC). A train-test split cohort with an inner leave-one-out strategy was performed to verify their conclusion. The final model used only six amino acids and achieved an excellent performance (AUC > 0.7 on all four stages) on discriminating lung cancer stages. They concluded that plasma amino acids (Proline, Ornithine, Arginine, etc.) might have the potential to become essential biomarkers for NSCLC. Our experimental results verified their conclusion and identified those amino acids as the top significant biomarkers listed in Supplementary Table S5. Our logistic regression model using the best five metabolites outperforms its discriminative AUC score on binary classification and is comparable to its performance on multivariate classification. In addition, Maeda et al*.* also explored the discrimination ability of plasma amino acid metabolites to distinguish cancer types^44^. Their final model has a similar performance to the logistic regression models in Section "Multivariate modeling: healthy control vs. adenocarcinomas vs. squamous-cell carcinoma vs. small-cell carcinoma" of our study.

Our experiment used the AUC scoring ranking algorithm based on metabolite classification ability to screen out other amino acids not mentioned in the above literatures. For example, 4-Oxoproline ranks the third most significant metabolite for discriminating lung cancer and the seventh most significant metabolite for discriminating lung cancer stages. 4-Hydroxyproline ranks the tenth most significant metabolite for discriminating lung cancer, tenth for lung cancer stages, and 13th for lung cancer types.

The result of pathway analysis and enrichment analysis based on KEGG of the differential metabolites in our study indicates that the pathway of aminoacyl-tRNA biosynthesis is significantly changed. The involving amino acids, such as l-Asparagine, l-Histidine, l-Phenylalanine, l-Serine, l-Methionine, l-Lysine, l-Leucine, l-Threonine, l-Tryptophan, and l-Proline, were detected, and their level was increased in patients. On the contrary, the levels of l-Arginine and l-Glutamine decreased in the plasma of lung cancer groups. In all, amino acids play an important role in tumor metabolism because they are necessary for tumor growth and proliferation^45^. We also observed that most amino acid concentrations increased in lung cancer tissue. For instance, Glutamine is a major nitrogen source and a carbon substrate for the synthesis of nucleotides and amino acids in cells. Our findings are in line with the previous studies^9,22,46–48^.

## Conclusions

Current research shows that the intensity distribution of some potential markers in cancer patients’ plasma is different from that of healthy subjects. We discovered several high-performance logistic regression models for diagnosis of cancer group, cancer stage, and cancer type. The five-metabolite-only classifiers used to distinguish cancer group always keeps the AUC performance greater than 87%. The AUC performance of the classifiers used to distinguish cancer stages is generally greater than 72%. The AUC performance of the classifier used to distinguish cancer types is greater than 75%. More-metabolite-involved classifiers demonstrated a more reliable AUC performance (close or higher than 90%). Our experimental results show that the metabolite-only multivariate classifier may be effective in distinguishing lung cancer patients, even for different stages and types. Although further data collection and quantitative experimental verification are necessary in the future, this method may be an effective and convenient screening tool for lung cancer patients. The classifier with several (less than 20) metabolites can be easily converted into a minimally invasive, high-performance, high-throughput, and low-cost lung cancer screening assay.

## Supplementary Information

Supplementary Information.
