# Supplementary material for: High-resolution metabolomic biomarkers for lung cancer diagnosis and prognosis
Source: Sci Rep. 2021 Jun 3;11:11805. doi: 10.1038/s41598-021-91276-2 (PMC8175557; doi:10.1038/s41598-021-91276-2)
Supplement: Supplementary file 1 — Supplementary Information. [file 41598_2021_91276_MOESM1_ESM.pdf]

Supplementary Materials

High-resolution Metabolomic Biomarkers for Lung Cancer Diagnosis and Prognosis

Shi-ang Qi[1,2], Qian Wu[3,4], Zhenpu Chen[2], Wei Zhang[1], Yongchun Zhou[2], Kaining Mao[1], Jia Li[2], Yuanyuan Li[3], Jie Chen[1], Youguang Huang[2], Yunchao Huang[2]

[1] Electrical and Computer Engineering, University of Alberta, Canada

[2] Third Affiliated Hospital of Kunming Medical University (Yunnan Cancer Hospital), Kunming, Yunnan 650118, China

[3] Shanghai Center for Bioinformation Technology & Shanghai Engineering Research Center of Pharmaceutical Translation, Shanghai Industrial Technology Institute, Shanghai 201203, China.

[4] Shanghai Fenglin Clinical Laboratory Co., Ltd, Shanghai 200231, China.

*Shi-ang Qi and Qian Wu contributed equally, and both are the first authors.

**Corresponding author: Jie Chen, Youguang Huang, and Yunchao Huang; jc65@ualberta.ca (Jie Chen); huangyouguang2008@126.com (Youguang Huang); huangyunchao2013@163.com (Yunchao Huang).

List of supplementary materials

Figure S1. The PCA score plots of metabolic profile of QC, health group and lung cancer group.
Figure S2. The OPLS-DA score plots and validation graphs of metabolic profile.
Figure S3. Pathway analysis of the differential metabolites between healthy controls and lung cancer patients
Figure S4. Enrichment analysis based on KEGG of the differential metabolites.
Figure S5. Enrichment analysis based on SMPDB of the differential metabolites.
Table S1. Qualitative identification results of differential plasma metabolites of lung cancer.
Table S2. The combined results of the core differential plasma metabolites of lung cancer.
Table S3. Performance of logistic regression models with various biomarkers for discriminating healthy controls, early-stage patients, and advanced-stage lung cancer patients.
Table S4. Performance of logistic regression models with various biomarkers for discriminating different lung cancer types.
Table S5. Summary of the top 20 significant differential metabolites of lung cancer.
A. Method: The detailed information of metabolomic analyses.
B. Multivariate Modeling: Healthy Control vs. Stage I & II Lung Cancer, Healthy Control vs. Stage III & IV Lung Cancer
C. Linear Relationship for the Logistic Regression Models

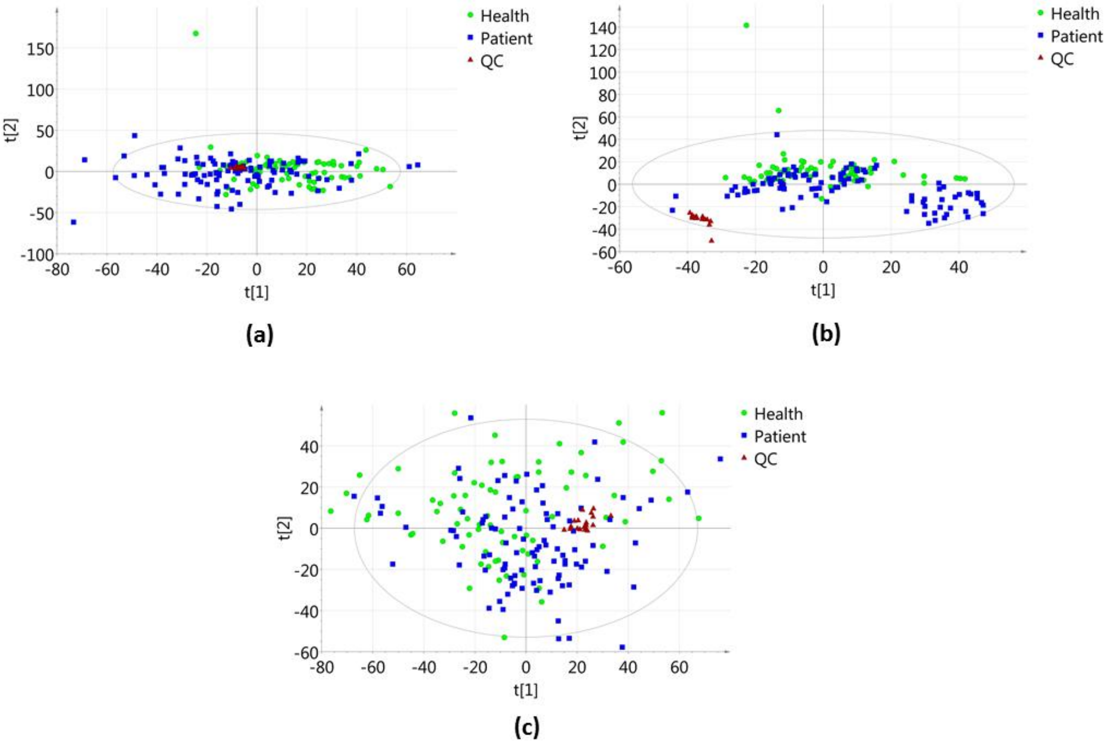

Figure S1. The PCA score plots of metabolic profile of QC, health group and lung cancer group. (a) RPLC-ESI− mode; (b) RPLC-ESI+ mode; and (c) HILIC-ESI+/− mode. Legends: brown triangles, QCs; green circles, healthy controls; blue boxes, lung cancer patients

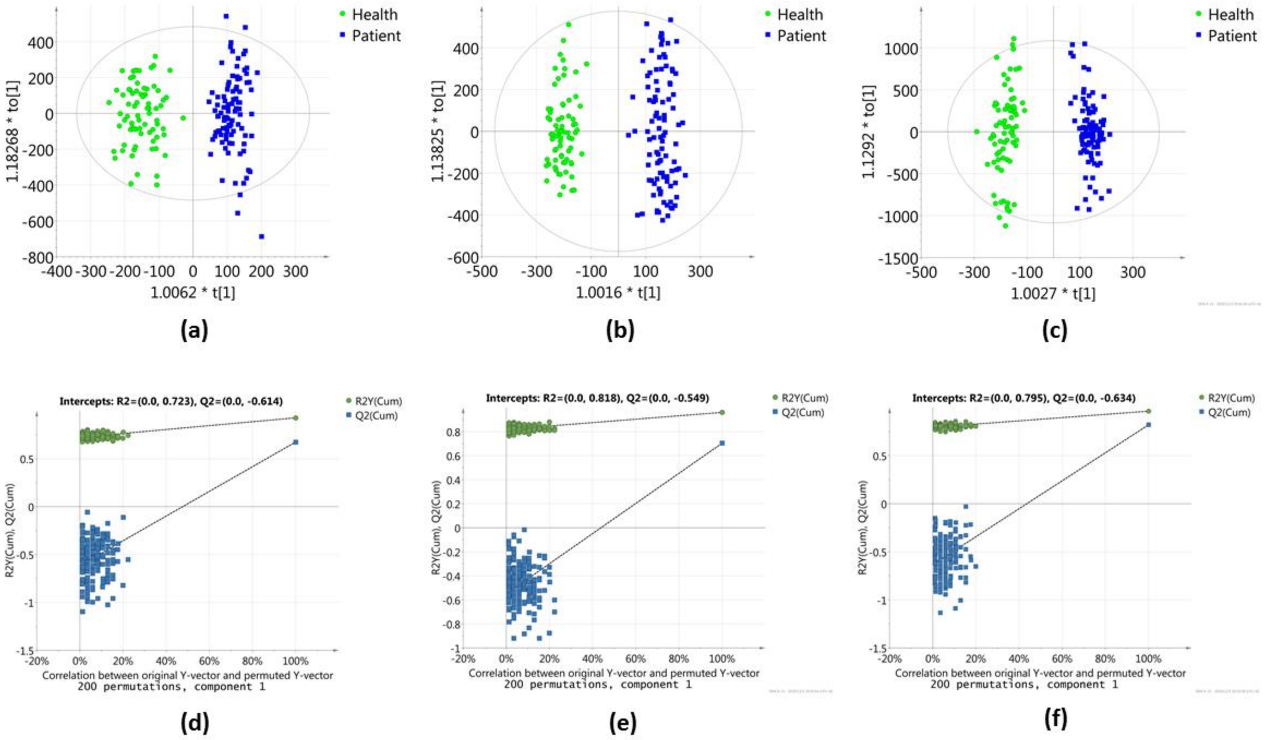

Figure S2. The OPLS-DA score plots and validation graphs of metabolic profile. (a) RPLC-ESI− mode; (b) RPLC-ESI+ mode; (c) HILIC-ESI+/− mode; (d), (e), and (f) are the corresponding validation models of the above models. Legends: green circles, healthy controls; blue boxes, lung cancer patients.

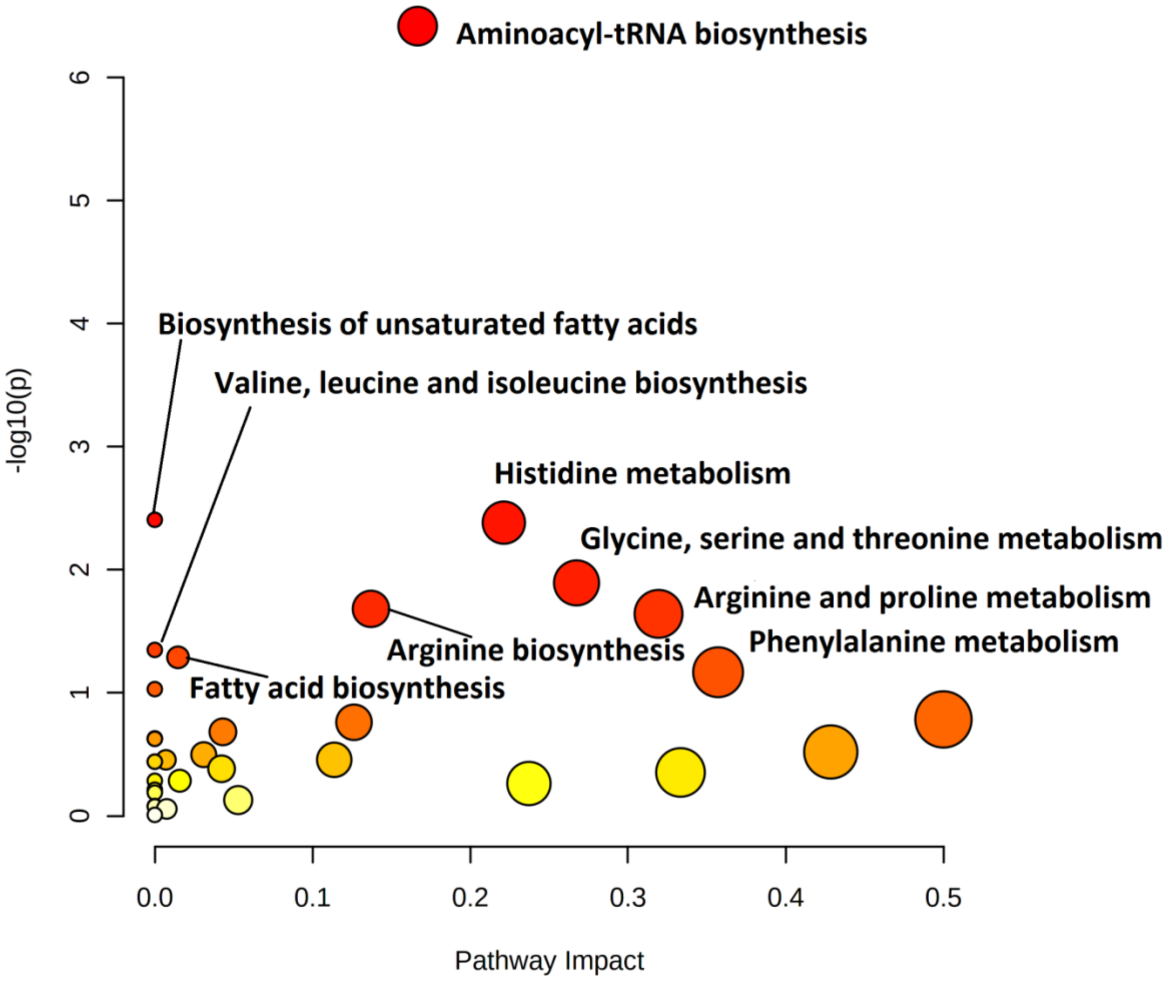

Figure S3. Pathway analysis of the differential metabolites between healthy controls and lung cancer patients using MetaboAnalyst 4.0 (http://www.metaboanalyst.ca/)

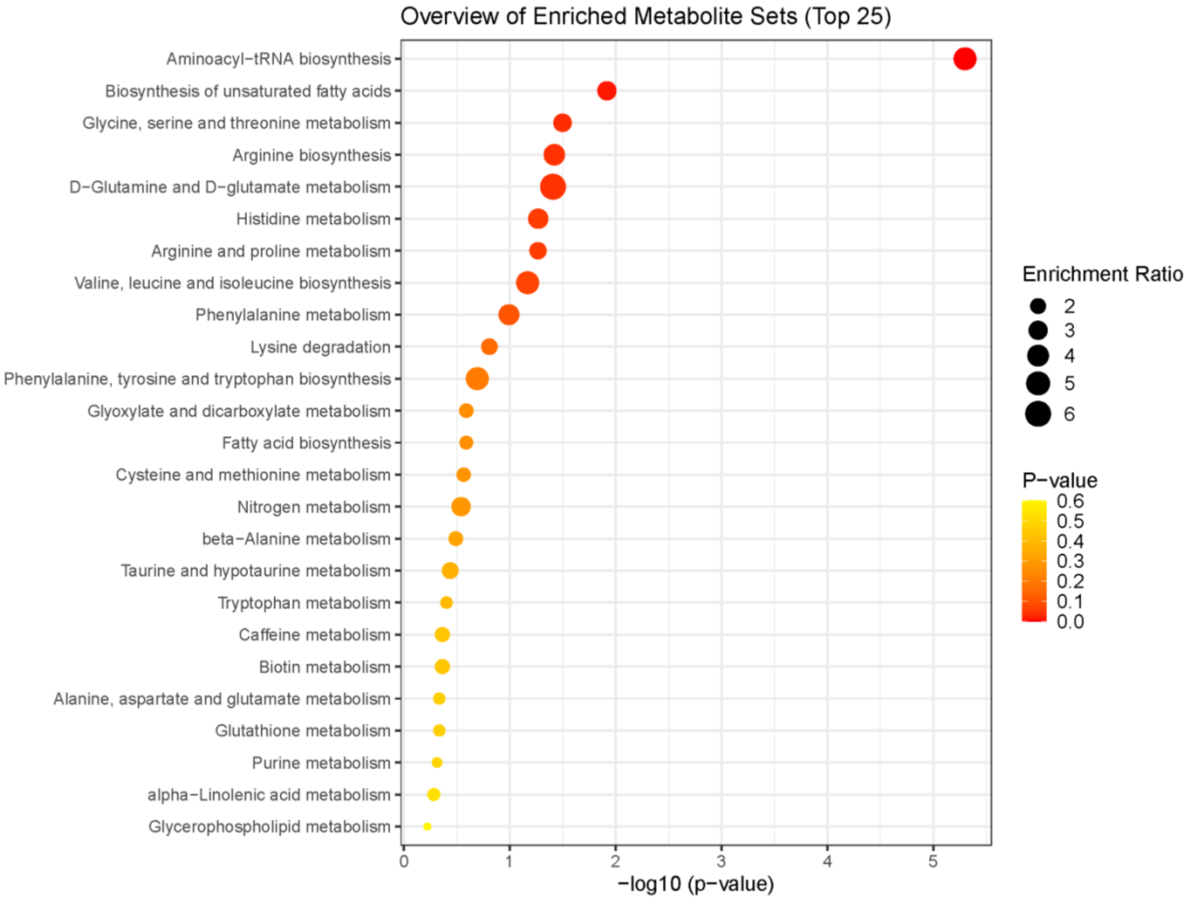

Figure S4. Enrichment analysis based on KEGG (Kyoto Encyclopedia of Genes and Genomes) of the differential metabolites between healthy controls and lung cancer patients using MetaboAnalyst 4.0 (http://www.metaboanalyst.ca/)

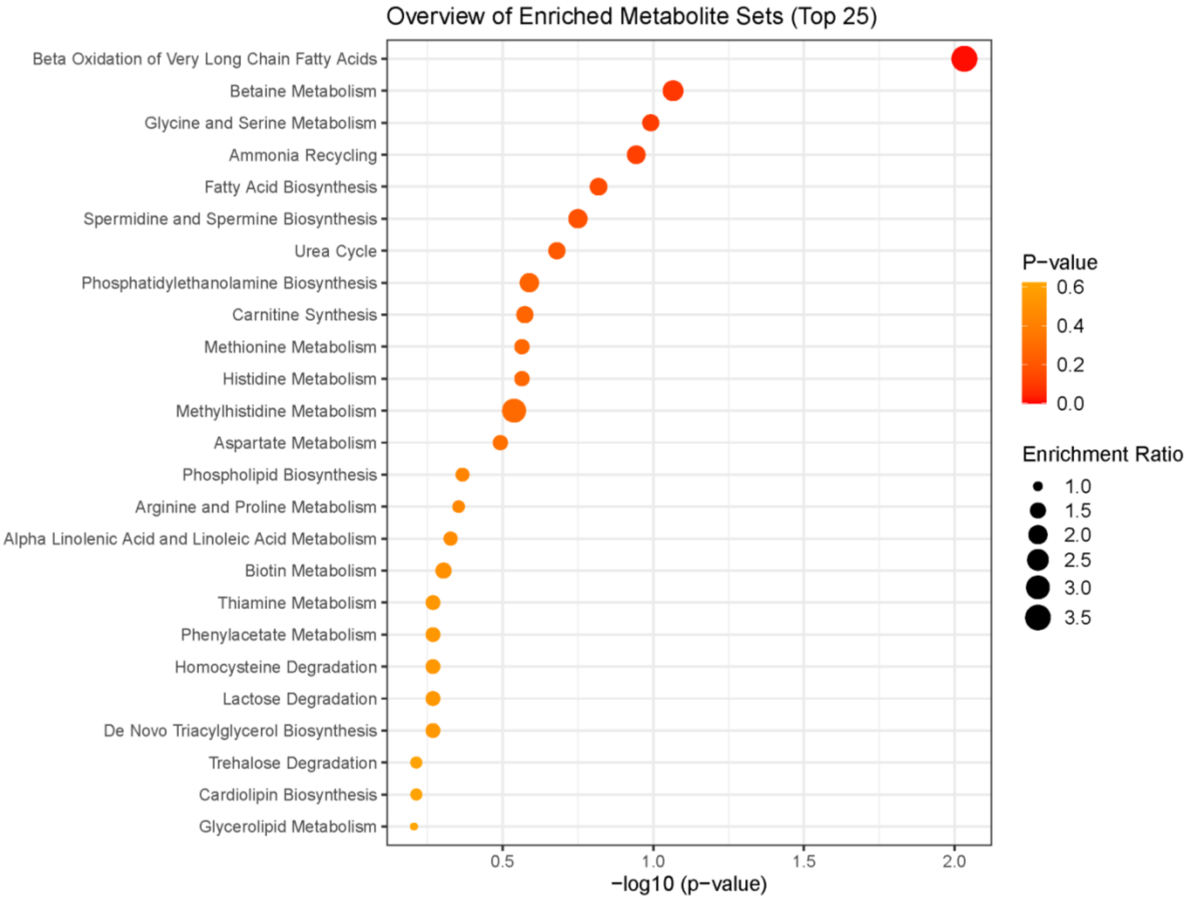

Figure S5. Enrichment analysis based on SMPDB (The Small Molecule Pathway Database) of the differential metabolites between healthy controls and lung cancer patients using MetaboAnalyst 4.0 (http://www.metaboanalyst.ca/)

Table S1. Qualitative identification results of differential plasma metabolites of lung cancer.

Mode	HMDB ID	Compound Name	M.W.	[M+H]+	[M-H]-	RT (min)	mzCloud	mzVault	VIP	FC	PV	Avg.H	Avg.P
RP Neg	HMDB0000115	Glycolic acid	76.01453		75.00725	0.90		86	1.89	1.28	1.23E-05	262.71	335.59
RP Neg	HMDB0000700	Hydroxypropionic acid	90.03008		89.0228	1.06		79	2.57	1.66	3.72E-10	149.80	248.02
RP Neg	HMDB0001429	Phosphate	97.96567		96.95839	0.87		83.9	1.08	1.11	2.46E-02	415.40	460.75
RP Neg	METPA0228	4-Oxoproline	129.0411		128.0338	0.90	64.3	80.8	2.60	1.33	1.86E-10	298.36	395.66
RP Neg	HMDB0000687	L-Leucine	131.0931		130.0858	1.01	70.4	80.8	1.33	1.35	1.10E-02	172.71	232.77
RP Neg	HMDB0000482	Caprylic acid	144.1136		143.1063	3.28	75.1		1.99	0.80	1.13E-02	618.12	495.08
RP Neg	HMDB0000208	Oxoglutaric acid	146.0205		145.0132	0.89			2.66	1.13	3.14E-02	2239.21	2521.50
RP Neg	HMDB0003011	O-Acetylserine	147.0517		146.0444	0.89			3.15	1.38	2.59E-12	348.48	480.09
RP Neg	HMDB0000177	L-Histidine	155.0682		154.0609	1.12			1.27	2.09	1.39E-04	35.83	74.84
RP Neg	HMDB0000847	Pelargonic acid	158.1294		157.1221	3.97	58.8		2.90	0.81	4.09E-04	986.60	798.23
RP Neg	HMDB0000849	Rhamnose	164.0677		163.0604	0.95			2.84	1.44	2.00E-05	369.53	532.99
RP Neg	HMDB0000511	Capric acid	172.1452		171.1379	4.56	75.6		1.35	0.83	4.45E-03	294.63	244.62
RP Neg	HMDB0001264	Dehydroascorbic acid	174.0153		173.008	0.89			1.24	1.28	5.06E-05	120.20	153.77
RP Neg	HMDB0033724	Undecylenic acid	184.1452		183.138	4.45			1.11	0.69	1.53E-05	76.22	52.97
RP Neg	HMDB0000947	Undecanoic acid	186.161		185.1537	5.12	76.2		1.71	0.75	8.62E-08	174.42	130.12
RP Neg	HMDB0000094	Citric acid	192.026		191.0187	0.88			2.79	1.13	2.17E-02	2282.59	2581.06
RP Neg	HMDB0000638	Dodecanoic acid	200.1767		199.1695	5.69	70.1		5.08	0.66	5.95E-09	1036.87	679.94
RP Neg	HMDB0028819	Glutamylglycine	204.0737		203.0664	0.89			1.27	2.40	2.21E-08	19.00	45.65
RP Neg	HMDB0000910	Tridecylic acid	214.1926		213.1853	6.29	57.7		2.05	0.74	6.59E-13	195.84	144.40
RP Neg	HMDB0000806	Myristic acid	228.2086		227.2013	14.52			1.85	1.53	1.24E-02	221.11	337.40
RP Neg	HMDB0000826	Pentadecanoic acid	242.2242		241.2169	7.58	80.5		3.69	0.80	1.56E-07	997.49	795.39
RP Neg	HMDB0000220	Palmitic acid	256.2399		255.2326	8.24	78.3	91.9	40.05	0.76	5.94E-14	72374.11	54925.69
RP Neg	No match	3-oxopalmitic acid	270.2195		269.2122	5.03			1.01	0.70	1.47E-08	51.05	35.88

RP Neg	HMDB0002259	Heptadecanoic acid	270.2559		269.2486	8.91		87.5	6.02	0.68	5.28E-12	1310.78	897.00
RP Neg	No match	Juniperic acid	272.2352		271.2279	4.94			1.14	0.62	1.82E-12	39.89	24.79
RP Neg	HMDB0000207	Oleic acid	282.2559		281.2486	8.72	82.7	96.7	1.82	0.86	2.09E-02	880.44	760.02
RP Neg	HMDB0000827	Stearic acid	284.2714		283.2641	9.56	82.2	88.3	29.93	0.75	2.19E-09	46140.02	34662.63
RP Neg	HMDB0000772	Nonadecanoic acid	298.2871		297.2798	10.19		84.5	1.42	0.68	1.57E-05	108.81	74.11
RP Neg	HMDB0002212	Arachidic acid	312.3029		311.2956	10.80	91.3	88.5	4.49	0.60	2.21E-06	794.96	474.86
RP Neg	HMDB0002183	Docosahexaenoic acid	328.2402		327.2329	7.22	70.2	74.7	1.98	0.82	8.75E-03	666.30	548.30
RP Neg	No match	3',5'-Cyclic IMP	330.037		329.0297	0.85			11.97	1.21	9.22E-07	12504.13	15069.34
RP Neg	HMDB0002833	Testosterone sulfate	368.1656		367.1583	2.38	75.6	77	2.27	0.71	3.59E-03	493.34	351.77
RP Neg	HMDB0000626	Deoxycholic acid	392.2927		391.2854	4.58	94.5		1.31	1.74	7.94E-03	80.62	140.07
RP Neg	HMDB0000708	Glycoursodeoxycholic acid	449.3141		448.3068	3.16	88		1.50	1.50	8.65E-03	146.72	220.54
RP Neg	HMDB0011507	LysoPE(18:2(9Z,12Z)/0:0)	477.2853		476.2781	6.00		85.6	1.03	1.16	4.20E-02	288.35	333.87
RP Neg	HMDB0011130	LysoPE(18:0/0:0)	481.3168		480.3095	7.64		82.5	1.05	1.22	9.00E-04	134.29	163.34
RP Neg	HMDB0004949	Cer(d18:1/16:0)	537.512		536.5047	13.23			1.01	1.63	1.10E-05	33.00	53.80
RP Neg	HMDB0010383	LysoPC(16:1(9Z)/0:0)	553.338		552.3308	5.77		82.5	1.13	1.25	2.29E-03	146.18	182.72
RP Neg	HMDB0010382	LysoPC(16:0/0:0)	555.3534		554.3461	6.61		83.6	4.40	1.11	1.52E-02	5947.82	6625.92
RP Neg	HMDB0002815	LysoPC(18:1(9Z)/0:0)	581.3691		580.3618	6.97		81.7	2.68	1.16	6.03E-03	1425.99	1650.94
RP Neg	HMDB0010384	LysoPC(18:0/0:0)	583.3849		582.3776	7.96		80.2	3.57	1.18	1.23E-02	2414.28	2845.90
RP Neg	HMDB0011128	LysoPC(0:0/18:0)	583.385		582.3777	7.63		82.5	1.49	1.20	1.37E-03	300.68	360.09
RP Neg	HMDB0010395	LysoPC(20:4(5Z,8Z,11Z,14Z)/0:0)	603.3536		602.3463	6.09		76	1.77	1.19	4.10E-03	494.02	587.55
RP Neg	HMDB0009789	PI(16:0/20:4(5Z,8Z,11Z,14Z))	858.5251		857.5178	10.49		66.7	1.04	1.28	1.35E-02	131.96	168.64
RP Neg	HMDB0009809	PI(18:0/18:2(9Z,1	862.5565		861.5492	11.14		63.9	1.63	1.28	1.30E-02	319.18	407.70

		2Z))											
RP Neg	HMDB0009815	PI(18:0/20:4(5Z,8Z,11Z,14Z))	886.5566		885.5493	11.06		66.3	4.13	1.36	3.79E-04	1125.97	1536.42
RP Pos	HMDB0000097	Choline	103.1003	104.1076		0.79	88.7	88.9	3.76	1.22	2.74E-08	1493.32	1819.47
RP Pos	HMDB0000562	Creatinine	113.0594	114.0666		0.81	89.1	88.6	2.98	0.89	5.30E-04	2808.67	2495.14
RP Pos	HMDB0000725	4-Hydroxyproline	131.0586	132.0659		0.88	51.8	69.6	2.38	0.79	3.21E-09	552.42	437.52
RP Pos	HMDB0000687	L-Leucine	131.0949	132.1022		0.91	83.2	88.5	5.76	0.93	1.50E-03	17317.31	16033.02
RP Pos	HMDB0000157	Hypoxanthine	136.0387	137.046		0.86	92.3	89.8	1.73	1.43	3.76E-08	164.27	234.96
RP Pos	HMDB0004827	Proline betaine	143.0949	144.1022		0.82	90.6		3.88	0.68	7.24E-03	2061.99	1400.68
RP Pos	HMDB0029737	Indole-3-carboxaldehyde	145.0531	146.0604		2.67	82.4	86.6	1.24	1.34	3.91E-04	172.29	230.77
RP Pos	HMDB0000895	Acetylcholine	145.1105	146.1178		0.82	50.7		1.30	1.23	1.72E-05	224.65	275.43
RP Pos	HMDB0000696	L-Methionine	149.0513	150.0586		0.91	86.4	90.1	2.38	0.91	1.14E-03	2414.25	2204.02
RP Pos	HMDB0034323	S-Allyl-L-cysteine	161.0514	162.0586		1.35			1.53	0.51	4.42E-15	72.73	37.21
RP Pos	HMDB0000721	Glycylproline	172.0851	173.0924		0.85			2.38	0.65	2.30E-04	522.25	340.56
RP Pos	HMDB0000517	L-Arginine	174.1121	175.1193		0.74	93.1	93.7	1.86	0.74	1.11E-06	325.54	241.60
RP Pos	HMDB0001046	Cotinine	176.0952	177.1025		0.90	70	78	1.58	0.38	6.47E-03	164.25	62.44
RP Pos	HMDB0000714	Hippuric acid	179.0588	180.066		2.90	66.2		1.12	0.46	3.07E-03	87.58	40.28
RP Pos	HMDB0000734	Indoleacrylic acid	187.0637	188.071		2.67	93.3		4.18	0.92	1.72E-02	10986.89	10096.17
RP Pos	HMDB0011173	Glycylhydroxyproline	188.08	189.0873		0.98			1.55	0.93	4.48E-02	2111.45	1963.98
RP Pos	HMDB0012286	Prenisteine	189.0829	190.0902		2.98			1.46	0.54	1.24E-18	65.65	35.29
RP Pos	HMDB0001847	Caffeine	194.0808	195.0881		2.91	96.9	85.3	4.39	0.29	5.19E-03	1023.15	292.90
RP Pos	HMDB0000201	L-Acetylcarnitine	203.1162	204.1234		0.87	93.2	93.4	3.26	0.92	4.41E-02	7732.86	7109.96
RP Pos	HMDB0000929	L-Tryptophan	204.0902	205.0974		2.67	92.1	93.8	4.29	0.92	1.45E-02	11004.71	10087.88
RP Pos	HMDB0001388	alpha-Linolenic acid	278.2251	279.2323		11.13	80.9		1.85	0.85	4.65E-03	949.51	806.21
RP Pos	HMDB0000305	Vitamin A	286.2301	287.2373		10.97			1.92	0.74	2.63E-03	550.97	405.18
RP Pos	No match	Acylcarnitine C8:0	287.2102	288.2175		5.00		89.2	2.01	0.82	2.85E-02	1229.88	1006.69
RP Pos	HMDB0010736	3-Oxooctadecanoi	298.2511	299.2584		11.15			1.76	0.57	1.11E-11	132.18	75.87

		c acid											
RP Pos	No match	Acylcarnitine C9:1	299.2103	300.2175		5.01		82.6	1.16	0.73	1.27E-04	149.12	108.47
RP Pos	No match	3-hydroxyoctanoylcarnitine	303.2051	304.2124		3.78			1.01	0.74	7.03E-04	136.85	101.46
RP Pos	HMDB0013645	N,N-Dimethylsphingosine	309.3036	310.3108		12.00	57.6		5.12	1.71	2.30E-07	924.88	1585.34
RP Pos	No match	Acylcarnitine C10:1	313.2255	314.2328		5.77		88.9	4.20	0.46	2.06E-03	1197.72	555.04
RP Pos	No match	Acylcarnitine C10:1	313.2258	314.2331		5.94		89.9	1.21	0.69	5.95E-03	190.74	131.65
RP Pos	HMDB0000651	Decanoylcarnitine	315.2416	316.2489		6.25	90.2	89.4	2.38	0.77	2.74E-02	1304.44	1000.84
RP Pos	No match	Acylcarnitine C12:1	341.2574	342.2647		6.99		89.5	1.35	0.70	3.06E-03	230.81	160.83
RP Pos	No match	Acylcarnitine C12:0	343.273	344.2802		7.51		88.8	1.26	0.73	2.23E-03	227.13	166.67
RP Pos	No match	Acylcarnitine C14:2	367.2731	368.2803		7.38		88.6	1.24	0.59	2.87E-05	105.69	61.97
RP Pos	No match	Acylcarnitine C14:1	369.2884	370.2957		8.26		89.1	1.30	0.74	5.79E-03	281.61	209.33
RP Pos	No match	Acylcarnitine C14:1	369.2887	370.296		8.05		87.2	1.13	0.80	9.66E-03	297.14	238.38
RP Pos	HMDB0000277	Sphingosine 1-phosphate	379.2496	380.2568		7.77			2.35	1.26	3.57E-09	454.14	574.01
RP Pos	HMDB0006461	Acylcarnitine C18:2	423.3357	424.343		9.40		85.1	4.57	1.75	6.06E-06	811.50	1416.73
RP Pos	No match	Glycochenodeoxycholic acid	449.315	450.3222		6.54			1.86	1.47	1.44E-02	373.46	550.55
RP Pos	HMDB0011128	LysoPC(0:0/18:0)	523.3648	524.372		9.98		81.8	1.98	1.14	1.96E-02	1458.12	1666.52
RP Pos	HMDB0010384	LysoPC(18:0/0:0)	523.3648	524.3721		10.22		74.6	4.73	1.10	2.73E-02	12028.17	13260.31
RP Pos	HMDB0000054	Bilirubin	584.264	585.2712		12.25	85.6		1.49	0.85	2.31E-02	782.12	661.63
HILIC	HMDB0000092	Dimethylglycine	103.0638	104.0711	102.0565	4.64			2.07	0.73	7.62E-03	361.37	262.61

HILIC	HMDB0000097	Choline	103.1002	104.1074	102.0929	4.50	93.9	90.8	7.91	1.13	3.97E-05	7792.09	8827.39
HILIC	HMDB0000187	L-Serine	105.0408	106.0481	104.0335	7.53		93	1.21	1.14	9.45E-04	219.62	250.42
HILIC	HMDB0000162	L-Proline	115.0616	116.0688	114.0543	4.91		79.8	2.07	1.11	1.48E-02	1042.82	1162.34
HILIC	HMDB0000043	Betaine	117.0792	118.0865	116.072	3.89	96.7	89.4	10.28	1.13	1.09E-03	16921.42	19101.12
HILIC	HMDB0000167	L-Threonine	119.0565	120.0637	118.0492	6.49		81.6	2.33	1.23	8.53E-05	411.61	506.42
HILIC	HMDB0000167	L-Threonine	119.0586	120.0659	118.0513	6.49	81.5	89.9	1.32	1.17	1.37E-03	220.02	256.86
HILIC	HMDB0000251	Taurine	125.0149	126.0222	124.0076	4.57	80	72.5	1.13	1.07	2.63E-02	529.65	568.79
HILIC	HMDB0002024	Imidazoleacetic acid	126.0432	127.0504	125.0359	4.67	75.6		1.20	1.15	1.25E-03	196.46	226.86
HILIC	METPA0228	4-Oxoproline	129.0408	130.0481	128.0336	4.19	51	63.4	2.57	1.61	1.30E-15	103.12	166.16
HILIC	METPA0228	4-Oxoproline	129.0409	130.0482	128.0336	3.38	63.9	81	3.75	1.42	2.65E-10	392.17	556.22
HILIC	HMDB0000070	Pipecolic acid	129.0791	130.0864	128.0718	13.89	87.4	88.9	2.15	1.09	2.60E-02	1514.47	1658.22
HILIC	HMDB0060460	cis-4-Hydroxy-D-proline	131.0583	132.0656	130.0511	11.35	58.3		3.47	0.86	2.66E-02	2591.94	2240.20
HILIC	HMDB0061880	N-Acetyl-beta-alanine	131.0585	132.0657	130.0512	10.99			2.68	1.74	1.45E-04	181.31	315.00
HILIC	HMDB0000064	Creatine	131.0697	132.077	130.0624	6.51	92.8	88.5	1.58	1.20	4.98E-02	431.78	520.29
HILIC	HMDB0000687	L-Leucine	131.0948	132.102	130.0875	5.57	70.8	71.7	1.25	0.45	6.02E-05	43.47	19.57
HILIC	HMDB0001645	L-Norleucine	131.0948	132.1021	130.0875	3.79	87.6	93.6	1.98	1.07	4.02E-02	1734.64	1861.14
HILIC	HMDB0000168	L-Asparagine	132.0518	133.059	131.0445	7.64	57.5	54.9	1.20	1.18	1.29E-04	148.39	175.09
HILIC	HMDB0000214	Ornithine	132.0882	133.0954	131.0809	13.57	57.8	57.5	1.65	1.33	6.88E-05	144.04	191.38
HILIC	HMDB0000214	Ornithine	132.0902	133.0975	131.0829	13.50	88.3	88.7	5.14	1.42	4.60E-10	738.45	1047.74
HILIC	HMDB0000943	Threonic acid	136.0368	137.044	135.0295	1.99			2.10	1.38	5.77E-07	167.13	230.75
HILIC	HMDB0000157	Hypoxanthine	136.0385	137.0458	135.0312	1.99	89	85.9	5.41	1.58	1.51E-10	579.14	916.77
HILIC	HMDB0002820	Methylimidazoleacetic acid	140.0588	141.066	139.0515	4.67	72.4		6.15	0.39	7.05E-05	932.57	363.79
HILIC	HMDB0240650	Ectoine	142.0744	143.0817	141.0672	6.03			1.17	0.76	3.47E-03	126.49	96.09
HILIC	HMDB0004827	Proline betaine	143.0947	144.102	142.0874	3.84	97.7		7.74	0.72	2.38E-02	6274.29	4512.59
HILIC	HMDB0000482	Caprylic acid	144.1133	145.1206	143.1061	1.20	60.1		1.23	0.63	4.49E-04	69.35	43.49
HILIC	HMDB0000895	Acetylcholine	145.1105	146.1177	144.1032	7.79	88.6		2.43	1.16	1.04E-04	641.28	745.82
HILIC	HMDB0000641	L-Glutamine	146.0694	147.0766	145.0621	4.64	58.4		1.07	0.86	5.38E-04	152.43	130.97
HILIC	HMDB0000182	L-Lysine	146.1057	147.113	145.0984	13.91	84.8	74.3	2.50	1.10	1.88E-02	1830.61	2014.57

HILIC	HMDB0000292	Xanthine	152.0317	153.039	151.0244	2.43	78.3		1.62	1.41	5.30E-06	97.91	137.81
HILIC	HMDB0000177	L-Histidine	155.0685	156.0757	154.0612	7.50	68.6	90.8	3.61	1.12	5.92E-03	2761.65	3101.23
HILIC	HMDB0000177	L-Histidine	155.0701	156.0773	154.0628	7.50	92.7	92	1.73	1.10	2.70E-02	1010.59	1106.92
HILIC	HMDB0000847	Pelargonic acid	158.1291	159.1364	157.1218	0.91			1.18	0.84	3.58E-04	159.18	133.57
HILIC	HMDB0002038	N(6)-Methyllysine	160.1196	161.1268	159.1123	11.23		60.9	1.31	0.84	4.10E-03	252.30	211.70
HILIC	HMDB0002038	N(6)-Methyllysine	160.1197	161.1269	159.1124	11.35		60.9	1.86	0.78	4.38E-07	219.28	171.28
HILIC	HMDB0000062	L-Carnitine	161.1053	162.1126	160.098	6.65	97	94.2	8.75	1.17	2.17E-06	6628.73	7767.69
HILIC	HMDB0002070	4-Hydroxy-2-oxo glutaric acid	162.0149	163.0222	161.0077	1.62			1.37	0.84	8.40E-03	304.06	255.16
HILIC	HMDB0000897	7-Methylguanine	165.0652	166.0725	164.0579	2.14	91.1		1.09	1.23	6.27E-05	85.09	104.99
HILIC	HMDB0000159	L-Phenylalanine	165.0775	166.0847	164.0702	3.34	70.3	93.1	2.37	1.09	1.18E-02	1792.24	1946.52
HILIC	HMDB0059750	D-Xylonic acid	166.0463	167.0536	165.039	4.80			1.10	0.80	5.31E-03	146.95	117.73
HILIC	HMDB0000001	1-Methylhistidine	169.0852	170.0925	168.0779	7.89	57.9	55.1	1.04	0.65	3.43E-02	91.16	58.85
HILIC	HMDB0000721	Glycylproline	172.0832	173.0905	171.0759	4.50			5.78	0.36	4.45E-05	772.57	281.13
HILIC	HMDB0000721	Glycylproline	172.0844	173.0917	171.0772	4.67			7.18	0.37	4.61E-05	1205.59	448.46
HILIC	HMDB0000854	Formiminoglutamic acid	174.0645	175.0717	173.0572	4.66			1.67	1.71	3.30E-02	137.37	234.49
HILIC	HMDB0028854	Glycylvaline	174.0999	175.1072	173.0926	4.13		50.4	2.32	0.38	2.32E-05	124.45	47.68
HILIC	HMDB0034365	L-Theanine	174.1005	175.1078	173.0932	4.64			1.97	0.63	4.42E-03	225.31	142.44
HILIC	HMDB0003357	N2-Acetylornithine	174.1006	175.1079	173.0933	4.37			1.62	0.73	3.66E-03	216.22	158.22
HILIC	HMDB0000517	L-Arginine	174.11	175.1173	173.1027	13.68	52	69.1	1.09	0.87	7.49E-04	181.71	158.33
HILIC	HMDB0000517	L-Arginine	174.1117	175.119	173.1044	13.89	89.4	86.8	2.50	0.71	4.06E-03	487.53	344.06
HILIC	HMDB0013287	Ne,Ne dimethyllysine	174.1368	175.1441	173.1295	13.99			2.21	1.58	1.24E-06	126.40	199.33
HILIC	HMDB0013287	Ne,Ne dimethyllysine	174.1371	175.1444	173.1298	13.96			2.29	1.55	1.86E-05	159.89	247.27
HILIC	HMDB0001046	Cotinine	176.095	177.1023	175.0877	1.11	78.3	82.4	1.22	0.42	1.53E-02	65.28	27.36
HILIC	HMDB0001046	Cotinine	176.0951	177.1024	175.0878	1.22	71.5	81.4	1.26	0.54	2.48E-02	95.26	51.36
HILIC	HMDB0001087	5-Methylthioribos	180.0448	181.0521	179.0376	0.88			2.37	1.55	7.37E-04	227.30	352.62

		e											
HILIC	HMDB0006050	o-Tyrosine	181.0717	182.079	180.0645	4.44	55.4		1.42	0.47	4.41E-02	121.56	57.49
HILIC	HMDB0061890	Pyroglutamylglycine	186.0642	187.0715	185.0569	8.82			1.67	0.31	5.60E-08	45.02	13.95
HILIC	HMDB0011173	Glycylhydroxyproline	188.0798	189.0871	187.0725	8.82			1.83	0.44	5.51E-05	89.55	39.41
HILIC	HMDB0001325	N6,N6,N6-Trimethyl-L-lysine	188.1527	189.1599	187.1454	13.89	89.6		1.57	1.17	1.83E-03	332.20	388.61
HILIC	No match	11-Aminoundecanoic acid	201.173	202.1803	200.1657	1.59			1.98	0.35	1.98E-28	38.23	13.26
HILIC	HMDB0003334	Symmetric dimethylarginine	202.1433	203.1506	201.136	12.32	81.3	87.8	1.44	1.16	2.25E-02	397.78	461.68
HILIC	HMDB0000684	L-Kynurenine	208.0849	209.0922	207.0776	3.58	85.9	85.4	1.02	1.20	2.28E-04	98.88	118.98
HILIC	HMDB0002013	Butyrylcarnitine	231.1472	232.1544	230.1399	3.48		89.2	2.27	1.24	2.09E-02	669.86	832.36
HILIC	HMDB0001959	gamma-Aminobutyryllysine	231.1585	232.1657	230.1512	4.85			1.08	0.08	7.48E-10	12.32	0.94
HILIC	HMDB0029158	gamma-Glutamyl serine	234.0843	235.0916	233.0771	10.02			5.40	1.17	1.63E-03	3643.12	4254.91
HILIC	HMDB0000192	L-Cystine	240.0238	241.0311	239.0165	10.92	90.7	90.4	1.29	0.72	2.37E-07	78.43	56.13
HILIC	HMDB0013286	N-Undecanoylglycine	243.1836	244.1909	242.1763	0.80			1.40	2.07	4.50E-06	29.10	60.31
HILIC	HMDB0000767	Pseudouridine	244.0688	245.076	243.0615	3.22	71.3	90.8	1.11	1.13	6.80E-04	194.22	219.55
HILIC	HMDB0000296	Uridine	244.069	245.0762	243.0617	1.99	72.1	90.7	3.71	0.87	4.71E-04	1967.04	1708.26
HILIC	HMDB0028757	Aspartyl-Leucine	246.0853	247.0926	245.078	11.27			2.70	0.84	9.09E-04	932.57	784.80
HILIC	HMDB0000086	Glycerophosphocholine	257.1031	258.1104	256.0958	8.02			1.77	1.33	2.01E-08	124.05	164.71
HILIC	HMDB0002259	Heptadecanoic acid	270.2553	271.2626	269.248	0.82		80.7	2.01	0.73	1.67E-09	175.11	127.55
HILIC	HMDB0011738	N2-gamma-Glutamylglutamine	275.1117	276.119	274.1045	10.28		67.9	1.03	1.28	3.50E-05	66.93	85.45
HILIC	HMDB0030964	Linolenelaidic acid	278.226	279.2333	277.2187	1.26			2.30	0.82	1.00E-03	650.33	534.65

HILIC	No match	Acylcarnitine C8:2	283.1785	284.1858	282.1712	2.28		66.1	1.26	0.88	2.77E-04	230.56	201.76
HILIC	HMDB0000827	Stearic acid	284.2708	285.2781	283.2636	0.81	81.8	87.7	8.63	0.81	1.51E-05	6285.93	5085.12
HILIC	HMDB0061859	Methyl hexadecanoic acid	287.2824	288.2897	286.2752	0.86	73.2		1.69	0.36	1.83E-02	131.89	47.11
HILIC	No match	3-hydroxyoctanoylcarnitine	303.2047	304.2119	302.1974	2.89			1.52	0.80	9.19E-03	300.62	240.96
HILIC	No match	Acylcarnitine C12:0	343.2729	344.2802	342.2656	1.47		86.1	1.13	0.76	8.84E-03	128.83	97.56
HILIC	HMDB0000175	Inosinic acid	348.0483	349.0555	347.041	11.11			8.21	1.22	1.91E-02	8777.58	10683.41
HILIC	No match	N-LINOLEOYL-4-AMINOBUTYRIC ACID	365.2923	366.2996	364.285	0.81			3.07	0.45	1.41E-22	117.50	52.53
HILIC	No match	Acylcarnitine C14:2	367.2725	368.2797	366.2652	1.43		73.3	1.01	0.80	7.51E-03	123.89	98.88
HILIC	No match	N-OLEOYL-4-AMINOBUTYRIC ACID	367.3089	368.3162	366.3016	0.81			7.37	0.48	5.06E-23	734.86	354.82
HILIC	HMDB0001032	Dehydroepiandrosterone sulfate	368.165	369.1723	367.1577	0.76		59.5	2.32	0.70	2.66E-03	384.82	269.81
HILIC	No match	3-Hydroxyhexadecadienoylcarnitine	411.2983	412.3056	410.291	1.69			1.65	1.72	2.32E-04	73.71	126.96
HILIC	HMDB0006461	Acylcarnitine C18:2	423.3352	424.3425	422.3279	1.30		82.8	1.03	1.33	6.03E-07	46.27	61.42
HILIC	HMDB0000708	Glycoursodeoxycholic acid	449.3133	450.3206	448.306	1.66	79.1		2.86	1.52	5.65E-03	403.05	612.46
HILIC	HMDB0000708	Glycoursodeoxycholic acid	449.3134	450.3207	448.3061	1.71	79.1		3.56	1.56	2.56E-03	527.37	823.57

RT, retention time; VIP, variable important in projection; FC, the fold change, which was calculated as the average of lung cancer patients relative to that of the healthy controls; PV, *p*-value obtained from Student's *t*-test; Avg.H, average normalized intensity of healthy controls samples; Avg.P, average normalized intensity of lung cancer patients samples.

Table S2. The combined results of the core differential plasma metabolites of lung cancer.

Query	Match	HMDB	PubChem	KEGG	SMILES	Comment
Glycolic acid	Glycolic acid	HMDB0000115	757	C03547	C(C(=O)O)O	1
Hydroxypropionic acid	Hydroxypropionic acid	HMDB0000700	68152	C01013	C(CO)C(=O)O	1
Phosphate	Phosphate	HMDB0001429	57424078	C00009	[O-]P(=O)([O-])[O-]	1
4-Oxoproline	4-Oxoproline	METPA0228	NA	C01877	NA	1
L-Leucine	L-Leucine	HMDB0000687	6106	C00123	CC(C)C[C@@H](C(=O)O)N	1
Caprylic acid	Caprylic acid	HMDB0000482	379	C06423	CCCCCCCC(=O)O	1
Pelargonic acid	Pelargonic acid	HMDB0000847	8158	C01601	CCCCCCCCC(=O)O	1
Capric acid	Capric acid	HMDB0000511	2969	C01571	CCCCCCCCCC(=O)O	1
Undecanoic acid	Undecanoic acid	HMDB0000947	8180	C17715	CCCCCCCCCCC(=O)O	1
Dodecanoic acid	Dodecanoic acid	HMDB0000638	3893	C02679	CCCCCCCCCCCC(=O)O	1
Tridecanoic acid	Tridecanoic acid	HMDB0000910	656741	C17076	CCCCCCCCCCCCC(=O)O	1
Pentadecanoic acid	Pentadecanoic acid	HMDB0000826	13849	C16537	CCCCCCCCCCCCCCC(=O)O	1
Palmitic acid	Palmitic acid	HMDB0000220	985	C00249	CCCCCCCCCCCCCCCC(=O)O	1
Heptadecanoic acid	Heptadecanoic acid	HMDB0002259	10465	NA	CCCCCCCCCCCCCCCCC(=O)O	1
Oleic acid	Oleic acid	HMDB0000207	445639	C00712	CCCCCCCC/C=C\CCCCCCCC(=O)O	1
Stearic acid	Stearic acid	HMDB0000827	5281	C01530	CCCCCCCCCCCCCCCCCC(=O)O	1
Nonadecanoic acid	Nonadecanoic acid	HMDB0000772	12591	C16535	CCCCCCCCCCCCCCCCCCC(=O)O	1
Arachidic acid	Arachidic acid	HMDB0002212	10467	C06425	CCCCCCCCCCCCCCCCCCCC(=O)O	1
Docosahexaenoic acid	Docosahexaenoic acid	HMDB0002183	445580	C06429	CC/C=C\C/C=C\C/C=C\C/C=C\C/C=C\C/C=C\CCC(=O)O	1
Testosterone sulfate	Testosterone sulfate	HMDB0002833	119207	NA	C[C@]12CC[C@H]3[C@H]([C@@H]1CC[C@@H]2OS(=O)(=O)O)CCC4=CC(=O)CC[C@]34C	1
Deoxycholic acid	Deoxycholic acid	HMDB0000626	222528	C04483	C[C@H](CCC(=O)O)[C@H]1CC[C@@H]2[C@@]1([C@H](C[C@H]3[C@H]2CC[C@H]4[C@@]3(CC[C@H](C4)O)C)O)C	1
Glycoursodeoxycholic acid	Glycoursodeoxycholic acid	HMDB0000708	12310288	NA	C[C@H](CCC(=O)NCC(=O)O)[C@H]1CC[C@@H]2[C@@]1(CC[C@H]3[C@H]2[C@H](C[C@H]4[C@@]3(CC[C	1

					@H](C4)O)C)O)C	
LysoPE(18:2(9Z,12Z)/0:0)	LysoPE(18:2(9Z,12Z)/0:0)	HMDB0011507	52925130	NA	CCCCC/C=C\C/C=C\CCCCCCCC(=O)OC[C@H](COP(=O)(O)OCCN)O	1
LysoPE(18:0/0:0)	LysoPE(18:0/0:0)	HMDB0011130	9547068	C21484	CCCCCCCCCCCCCCCCCC(=O)OC[C@H](COP(=O)(O)OCCN)O	1
LysoPC(16:1(9Z))	LysoPC(16:1(9Z))	HMDB0010383	24779461	C04230	CCCCCC/C=C\CCCCCCCC(=O)OC[C@H](COP(=O)([O-])OCC[N+](C)(C)C)O	1
LysoPC(16:0)	LysoPC(16:0)	HMDB0010382	460602	C04230	CCCCCCCCCCCCCCCC(=O)OC[C@H](COP(=O)([O-])OCC[N+](C)(C)C)O	1
LysoPC(18:1(9Z))	LysoPC(18:1(9Z))	HMDB0002815	16081932	C04230	CCCCCCCC/C=C\CCCCCCCC(=O)OC[C@H](COP(=O)([O-])OCC[N+](C)(C)C)O	1
LysoPC(18:0)	LysoPC(18:0)	HMDB0010384	497299	C04230	CCCCCCCCCCCCCCCCCC(=O)OC[C@H](COP(=O)([O-])OCC[N+](C)(C)C)O	1
LysoPC(0:0/18:0)	LysoPC(0:0/18:0)	HMDB0011128	24779491	NA	CCCCCCCCCCCCCCCCCC(=O)O[C@H](CO)COP(=O)([O-])OCC[N+](C)(C)C	1
LysoPC(20:4(5Z,8Z,11Z,14Z))	LysoPC(20:4(5Z,8Z,11Z,14Z))	HMDB0010395	24779476	C04230	CCCCC/C=C\C/C=C\C/C=C\C/C=C\CCCC(=O)OC[C@H](COP(=O)([O-])OCC[N+](C)(C)C)O	1
Choline	Choline	HMDB0000097	305	C00114	C[N+](C)(C)CCO	1
L-Serine	L-Serine	HMDB0000187	5951	C00065	C([C@@H](C(=O)O)N)O	1
L-Proline	L-Proline	HMDB0000162	145742	C00148	C1C[C@H](NC1)C(=O)O	1
Betaine	Betaine	HMDB0000043	247	C00719	C[N+](C)(C)CC(=O)[O-]	1
L-Threonine	L-Threonine	HMDB0000167	6288	C00188	C[C@H]([C@@H](C(=O)O)N)O	1
Taurine	Taurine	HMDB0000251	1123	C00245	C(CS(=O)(=O)O)N	1
Imidazoleacetic acid	Imidazoleacetic acid	HMDB0002024	96215	C02835	C1=C(NC=N1)CC(=O)O	1
Pyroglutamic acid	Pyroglutamic acid	HMDB0000267	7405	C01879	C1CC(=O)N[C@@H]1C(=O)O	1
Pipecolic acid	Pipecolic acid	HMDB0000070	849	C00408	C1CCNC(C1)C(=O)O	1
4-Hydroxyproline	4-Hydroxyproline	HMDB0000725	5810	C01157	C1[C@H](CN[C@@H]1C(=O)O)O	1
Creatine	Creatine	HMDB0000064	586	C00300	CN(CC(=O)O)C(=N)N	1
L-Norleucine	L-Norleucine	HMDB0001645	21236	C01933	CCCC[C@@H](C(=O)O)N	1
L-Asparagine	L-Asparagine	HMDB0000168	6267	C00152	C([C@@H](C(=O)O)N)C(=O)N	1
Ornithine	Ornithine	HMDB0000214	6262	C00077	C(C[C@@H](C(=O)O)N)CN	1
Hypoxanthine	Hypoxanthine	HMDB0000157	790	C00262	C1=NC2=C(N1)C(=O)N=CN2	1

Methylimidazoleacetic acid	Methylimidazoleacetic acid	HMDB0002820	75810	C05828	CN1C=C(N=C1)CC(=O)O	1
Proline betaine	Proline betaine	HMDB0004827	7016563	C10172	C[N+]1(CCC[C@H]1C(=O)[O-])C	1
Acetylcholine	Acetylcholine	HMDB0000895	6060	C01996	CC(=O)OCC[N+](C)(C)C	1
L-Glutamine	L-Glutamine	HMDB0000641	5961	C00064	C(CC(=O)N)[C@@H](C(=O)O)N	1
L-Lysine	L-Lysine	HMDB0000182	5962	C00047	C(CCN)C[C@@H](C(=O)O)N	1
Xanthine	Xanthine	HMDB0000292	1188	C00385	C1=NC2=C(N1)C(=O)NC(=O)N2	1
L-Histidine	L-Histidine	HMDB0000177	6274	C00135	C1=C(NC=N1)C[C@@H](C(=O)O)N	1
N(6)-Methyllysine	N(6)-Methyllysine	HMDB0002038	164795	C02728	CNCCCC[C@@H](C(=O)O)N	1
L-Carnitine	L-Carnitine	HMDB0000062	2724480	C00318	C[N+](C)(C)C[C@H](CC(=O)[O-])O	1
7-Methylguanine	7-Methylguanine	HMDB0000897	11361	C02242	CN1C=NC2=C1C(=O)N=C(N2)N	1
L-Phenylalanine	L-Phenylalanine	HMDB0000159	6140	C00079	C1=CC=C(C=C1)C[C@@H](C(=O)O)N	1
1-Methylhistidine	1-Methylhistidine	HMDB0000001	92105	C01152	CN1C=C(N=C1)C[C@@H](C(=O)O)N	1
Glycyl-Valine	Glycyl-Valine	HMDB0028854	97417	NA	CC(C)C(NC(=O)CN)C(O)=O	1
L-Arginine	L-Arginine	HMDB0000517	6322	C00062	C(C[C@@H](C(=O)O)N)CN=C(N)N	1
Cotinine	Cotinine	HMDB0001046	408	NA	CN1C(CCC1=O)C2=CN=CC=C2	1
o-Tyrosine	o-Tyrosine	HMDB0006050	91482	NA	C1=CC=C(C(=C1)CC(C(=O)O)N)O	1
N6,N6,N6-Trimethyl-L-lysine	N6,N6,N6-Trimethyl-L-lysine	HMDB0001325	440120	C03793	C[N+](C)(C)CCCC[C@@H](C(=O)[O-])N	1
Symmetric dimethylarginine	Symmetric dimethylarginine	HMDB0003334	169148	NA	CNC(=NC)NCCC[C@@H](C(=O)O)N	1
L-Kynurenine	L-Kynurenine	HMDB0000684	161166	C00328	C1=CC=C(C(=C1)C(=O)C[C@@H](C(=O)O)N)N	1
Myristic acid	Myristic acid	HMDB0000806	11005	C06424	CCCCCCCCCCCCCC(=O)O	1
Butyrylcarnitine	Butyrylcarnitine	HMDB0002013	439829	C02862	CCCC(=O)OC(CC(=O)[O-])C[N+](C)(C)C	1
L-Cystine	L-Cystine	HMDB0000192	67678	C00491	C([C@@H](C(=O)O)N)SSC[C@@H](C(=O)O)N	1
Pseudouridine	Pseudouridine	HMDB0000767	15047	C02067	C1=C(C(=O)NC(=O)N1)[C@H]2[C@@H]([C@@H]([C@H](O2)CO)O)O	1
Uridine	Uridine	HMDB0000296	6029	C00299	C1=CN(C(=O)NC1=O)[C@H]2[C@@H]([C@@H]([C@H](O2)CO)O)O	1
N2-gamma-Glutamylglutamine	N2-gamma-Glutamylglutamine	HMDB0011738	150914	C05283	C(CC(=O)N[C@@H](CCC(=O)N)C(=O)O)[C@@H](C(=O)O)N	1
Dehydroepiandrosteron	Dehydroepiandroster	HMDB0001032	12594	C04555	C[C@]12CC[C@H]3[C@H]([C@@H]1CCC2=O)CC=C4[	1

e sulfate	one sulfate				C@@]3(CC[C@@H](C4)OS(=O)(=O)O)C	
Linoelaidyl carnitine	Linoelaidyl carnitine	HMDB0006461	53477834	NA	CCCCC/C=C/C/C=C/CCCCCCCC(=O)OC(CC(=O)[O-])C[N+](C)(C)C	1
Creatinine	Creatinine	HMDB0000562	588	C00791	CN1CC(=O)N=C1N	1
Inosine	Inosine	HMDB0000195	6021	C00294	C1=NC(=O)C2=C(N1)N(C=N2)[C@H]3[C@@H]([C@@H]([C@H](O3)CO)O)O	1
L-Methionine	L-Methionine	HMDB0000696	6137	C00073	CSCC[C@@H](C(=O)O)N	1
Hippuric acid	Hippuric acid	HMDB0000714	464	C01586	C1=CC=C(C=C1)C(=O)NCC(=O)O	1
Indoleacrylic acid	Indoleacrylic acid	HMDB0000734	15030923	NA	C1=CC=C2C(=C1)C=C(N2)/C=C/C(=O)O	1
Caffeine	Caffeine	HMDB0001847	2519	C07481	CN1C=NC2=C1C(=O)N(C(=O)N2C)C	1
L-Acetylcarnitine	L-Acetylcarnitine	HMDB0000201	7045767	C02571	CC(=O)OC(CC(=O)[O-])C[N+](C)(C)C	1
L-Tryptophan	L-Tryptophan	HMDB0000929	6305	C00078	C1=CC=C2C(=C1)C(=CN2)C[C@@H](C(=O)O)N	1
Alpha-Linolenic acid	Alpha-Linolenic acid	HMDB0001388	5280934	C06427	CC/C=C\C/C=C\C/C=C\CCCCCCCC(=O)O	1
N,N-Dimethylsphingosine	N,N-Dimethylsphingosine	HMDB0013645	5282309	C13914	CCCCCCCCCCCCC/C=C/[C@H]([C@H](CO)N(C)C)O	1
Decanoylcarnitine	Decanoylcarnitine	HMDB0000651	10245190	NA	CCCCCCCCCC(=O)OC(CC(=O)[O-])C[N+](C)(C)C	1
Bilirubin	Bilirubin	HMDB0000054	21252250	C00486	CC1=C(NC(=C1CCC(=O)O)CC2=C(C(=C(N2)/C=C\3/C(=C(C(=O)N3)C)C=C)C)CCC(=O)O)/C=C\4/C(=C(C(=O)N4)C=C)C	1

Table S3. Performance of logistic regression models with various biomarkers for discriminating healthy controls, early-stage patients, and advanced-stage lung cancer patients.

	**Top 5 significant metabolites**		**Top 10 significant metabolites**		**Top 20 significant metabolites**	
	**Discovery**	**Validation**	**Discovery**	**Validation**	**Discovery**	**Validation**
**AUC (healthy controls)**	0.910 (+/- 0.091)	0.858	0.961 (+/- 0.069)	0.914	0.989 (+/- 0.019)	0.939
**AUC (early-stage patients)**	0.718 (+/- 0.211)	0.717	0.733 (+/- 0.134)	0.731	0.715 (+/- 0.190)	0.794
**AUC (advanced-stage patients)**	0.751 (+/- 0.161)	0.759	0.793 (+/- 0.119)	0.793	0.753 (+/- 0.224)	0.815
**Accuracy**	0.599 (+/- 0.143)	0.536	0.647 (+/- 0.194)	0.551	0.626 (+/- 0.228)	0.667
**Precision**	0.613 (+/- 0.213)	0.547	0.634 (+/- 0.256)	0.515	0.625 (+/- 0.257)	0.648
**Recall**	0.599 (+/- 0.143)	0.536	0.647 (+/- 0.194)	0.551	0.626 (+/- 0.228)	0.667

Table S4. Performance of logistic regression models with various biomarkers for discriminating different lung cancer types.

	**Top 5 significant metabolites**	**Top 10 significant metabolites**	**Top 20 significant metabolites**
**AUC (healthy control)**	0.891	0.916	0.973
**AUC (ADC patients)**	0.758	0.818	0.895
**AUC (SCC patients)**	0.746	0.810	0.827
**AUC (SCLC patients)**	0.826	0.754	0.872
**Accuracy**	0.731	0.731	0.826
**Precision**	0.651	0.691	0.819
**Recall**	0.731	0.731	0.826

Table S5. Summary of the top 20 significant differential metabolites of lung cancer

Group					Stage					Type				
Metabolite Name	Trend	AUC	P-value (Student t-test)	P-value (Mann-Whitney U test)	Metabolite Name	Trend	AUC	P value (one-way ANOVA test)	P value (Kruskal-Wallis H-test)	Metabolite Name	Trend	AUC	P value (one-way ANOVA test)	P value (Kruskal-Wallis H-test)
Palmitic acid[(a)]	↓	0.860	5.94E-14	7.81E-16	Palmitic acid[(a)]	↓	0.767	8.08E-16	1.99E-14	Palmitic acid[(a)]	↓	0.783	3.99E-15	5.62E-14
Heptadecanoic acid[(a)]	↓	0.841	5.28E-12	1.90E-14	Heptadecanoic acid[(a)]	↓	0.747	5.02E-14	6.56E-13	Heptadecanoic acid[(a)]	↓	0.750	8.86E-13	3.75E-12
4-Oxoproline[(c)]	↑	0.833	2.65E-10	2.69E-11	Ornithine[(c)]	↑	0.734	2.80E-09	1.71E-10	Ornithine[(c)]	↑	0.736	1.22E-07	8.76E-10
Tridecanoic acid[(a)]	↓	0.811	6.59E-13	1.48E-12	Tridecanoic acid[(a)]	↓	0.727	4.08E-13	7.33E-12	Pentadecanoic acid[(a)]	↓	0.694	1.68E-05	1.04E-05
Ornithine[(c)]	↑	0.807	4.60E-10	9.74E-12	Stearic acid[(a)]	↓	0.720	5.01E-11	2.51E-10	Acylcarnitine C8:1[(b)]	↓	0.691	1.67E-04	8.81E-06
Pentadecanoic acid[(a)]	↓	0.799	1.56E-07	9.27E-11	Hypoxanthine[(c)]	↑	0.716	2.85E-11	3.56E-10	Stearic acid[(a)]	↓	0.690	6.27E-10	3.82E-09
Hypoxanthine[(c)]	↑	0.794	1.51E-10	5.82E-10	4-Oxoproline[(c)]	↑	0.712	8.63E-09	3.29E-10	Dodecanoic acid[(a)]	↓	0.690	3.27E-09	3.29E-10
Stearic acid[(a)]	↓	0.788	2.19E-09	3.66E-11	Dodecanoic acid[(a)]	↓	0.711	2.71E-09	1.62E-10	N(6)-Methyllysine[(c)]	↑	0.688	2.52E-06	1.33E-06
Caprylic acid[(c)]	↓	0.775	4.49E-04	1.20E-10	Pentadecanoic acid[(a)]	↓	0.710	2.31E-08	7.02E-10	N,N-Dimethylsphingosine[(c)]	↑	0.687	3.61E-07	3.66E-05
4-Hydroxyproline[(b)]	↓	0.772	3.21E-09	1.98E-09	4-Hydroxyproline[(b)]	↓	0.695	4.01E-09	1.87E-08	Acylcarnitine C14:3[(b)]	↓	0.685	4.46E-04	3.87E-05
Dodecanoic acid[(a)]	↓	0.768	5.95E-09	2.43E-11	N(6)-Methyllysine[(c)]	↑	0.687	7.54E-07	9.28E-08	Acylcarnitine C10:2[(b)]	↓	0.681	4.21E-07	3.91E-07
Acylcarnitine C10:2[(b)]	↓	0.765	6.29E-08	1.54E-08	L-Cystine[(c)]	↓	0.687	1.41E-06	3.63E-08	N2,N2-Dimethylguanosine[(c)]	↑	0.680	3.24E-08	3.65E-08
N2,N2-Dimethylguanosine[(c]	↑	0.753	2.98E-08	1.21E-08	Acylcarnitine C10:2[(b)]	↓	0.684	1.95E-07	2.40E-07	4-Hydroxyproline[(b)]	↓	0.678	2.35E-09	1.50E-08

)														
Hydroxypropionic acid[a]	↑	0.745	3.72E-10	1.03E-08	Caprylic acid[c]	↓	0.682	3.95E-04	6.46E-10	Myristic acid[a]	↓	0.678	2.86E-03	5.89E-04
N(6)-Methyllysine[c]	↑	0.745	4.38E-07	3.53E-08	N2,N2-Dimethylguanosine[c]	↑	0.678	3.15E-07	2.33E-07	Biliverdin[b]	↓	0.673	3.62E-03	3.25E-05
L-Cystine[c]	↓	0.739	2.37E-07	2.81E-09	Acylcarnitine C14:2[b]	↓	0.677	6.31E-05	1.06E-06	L-Kynurenine[c]	↑	0.668	1.31E-05	3.08E-05
Choline[b]	↑	0.736	2.74E-08	4.48E-08	Undecanoic acid[a]	↓	0.676	2.46E-08	8.27E-08	Acylcarnitine C12:1[b]	↓	0.665	1.38E-04	1.08E-04
Xanthine[c]	↑	0.735	5.30E-06	7.28E-08	N6-Acetyl-L-lysine[c]	↑	0.674	1.62E-05	2.46E-06	Alpha-Linolenic acid[a]	−	0.665	5.12E-03	2.97E-04
Arachidic acid[a]	↓	0.731	2.21E-06	3.04E-07	Hydroxypropionic acid[a]	↑	0.673	4.01E-09	9.85E-08	5-Hydroxyindoleacetic acid[c]	↑	0.664	1.85E-05	4.73E-05
L-Arginine[b]	↓	0.729	1.11E-06	1.50E-07	Choline[b]	↑	0.671	4.14E-07	6.25E-07	Hydroxypropionic acid[a]	↑	0.663	2.32E-07	2.30E-06

Metabolite Name, (a) RPLC Neg, (b) RPLC Pos, and (c) HILIC; Trend, average normalized intensity of lung cancer patients compared with the controls.

A. Methods

The detailed information of metabolomic analyses

LC-MS and LC-MS/MS

An Ultimate-3000 UPLC system coupled to a Q Exactive hybrid quadrupole-Orbitrap MS system (Thermo Scientific) was used for the sample analysis. Before injection, the residues were resuspended in platform-specific solutions. A combination of three conditions: (A) Hydrophilic interaction liquid chromatography (HILIC)-MS with ESI+/ESI− switching mode; (B) Reversed-phase liquid chromatography (RPLC)-MS with ESI+ mode; and (C) RPLC-MS with ESI− mode, was used for the untargeted metabolomic analysis. The obtained residue was prepared in 100 μL of acetonitrile/water (1:1, v/v) for the HILIC-MS analysis, and 2-chloro-L-phenylalanine was used as an internal standard. The residue was made in 100 μL of 80% methanol for the RPLC-MS analysis, and 2-Chloro-L-phenylalanine, hexanoyl-L-carnitine-(N-methyl-d3), lysophosphatidylcholine 12:0, and nonadecanoic-d37 acid were used as internal standards.

Gradient Profile for HILIC-MS Method: An Acquity UPLC BEH Amide column (1.7 μm × 2.1 mm × 100 mm, Waters) with a VanGuard pre-column was used. The column temperature was set at 40°C. The mobile phases were 20mM ammonium acetate and 20mM ammonium hydroxide in water/acetonitrile (95:5, v:v) (A) and acetonitrile (B). The flow rate was 0.35 mL/min and the injection volume was 5 μL. The gradient was 85% B in 0–1.0 min, 85–65% B in 1.0–12.0 min, 65–40% B in 12.0–12.1 min, 40% B in 12.1–15.0 min, 40–85% B in 15.0–15.1 min, and 85% B in 15.1–20.0 min. All the samples were kept at 4°C during the analysis.

Gradient Profiles for RPLC-ESI+/MS Methods: A Hypersil GOLD C18 column (1.9 μm × 2.1 mm × 100 mm, Thermo Scientific) with a guard filter was used. The column temperature was set at 45°C. For ESI+ mode, the mobile phases were 2% water in acetonitrile with 0.1% formic acid (A) and 5% acetonitrile in water with 0.1% formic acid (B). The flow rate was 0.30 mL/min and the injection volume was 5 μL. The gradient was 100% B in 0–1.5 min, 100–80% B in 1.5–10.0 min, 80–3% B in 10.0–14.5 min, 3–100% B in 14.5–14.6 min, 100% B in 14.6–18.0 min. All the samples were kept at 4°C during the analysis.

Gradient Profiles for RPLC-ESI–/MS Methods: A Hypersil GOLD C8 column (1.9 μm × 2.1 mm × 100 mm, Thermo Scientific) with a guard filter was used. The column temperature was set at 45°C. The mobile phases were 20% isopropanol in acetonitrile (A) and 5% acetonitrile in water with 10 mmol ammonium acetate (pH was adjusted to 5.0 by acetic acid) (B). The flow rate was 0.30 mL/min and the injection volume was 5 μL. The gradient was 70% B in -3.5–0.0 min, 70–40% B in 0.0–3.0 min, 40–0% B in 3.0–12.5 min, 0% B in 12.5–16.0 min, and 0–70% B in 16.0–16.5 min. All the samples were kept at 4°C during the analysis.

Electrospray ionization in both of the liquid condition was carried out using the following conditions. Full scan data was acquired in positive, negative, or positive/negative ion switching modes in profile mode at 70,000 resolution (at m/z range 70–1050), an automatic gain control target of 3e6, with spray voltages +3.0 kV and –2.5 kV. The capillary temperature was set at 350°C with the sheath gas flow rate at 45 arb and aux gas flow rate at 15 arb. The heater temperature was set at 350°C. The S-Lens RF level was set at 50.

The MS/MS was operated at a resolving power of 70,000 in full-scan mode (scan range: 70-1050 m/z); automatic gain control target: 1e6 both in positive and negative ion mode) and of 17,500 in the Top10 data-dependent MS2 mode (stepped normalized collision energy: 15, 30 and 45 in all positive mode and negative mode for RPLC-ESI, while 30, 45, 60 in negative ion mode for HILIC method; Injection time: 100 ms; Isolation window: 4.0 m/z; automatic gain control target: 2e5) with dynamic exclusion setting of 6.0 s.

Data Preprocessing

MS raw data were acquired using the software Xcalibur (version 3.1, Thermo Scientific). The spectra selection, retention time alignment, and peak identification were performed using Compound Discoverer (version 3.0, Thermo Scientific), to obtain the data matrix containing molecular weight, retention time, peak intensity and annotation result. A workflow named "Untargeted Metabolomics with statistics detect unknowns with ID using Online Database and mzLogic" was chosen to process. Key processing parameters were: Mass Tolerance, 10 ppm; RT Tolerance, 0.2 min for RPLC and 0.5 min for HILIC; Max Peak Width, 0.5 min for RPLC and 0.6 min for HILIC; Min Peak Intensity, 500,000. The metabolites were identified by exact masses and fragmentation spectra with databases such as

mzCloud, ChemSpider, LipidBlast and Fiehn HILIC database. The free fatty acids were further identified by reference standards. A support vector regression-based normalization method [Normalization and integration of large-scale metabolomics data using support vector regression. Metabolomics, 2016, 12:89] was then used to reduce unwanted variations in such a large-scale metabolomics study.

The normalized dataset was then imported into SIMCA (V15.0, Umetrics, Umeå, Sweden) for further multivariate statistical analysis [Urinary metabolomic study of non-small cell lung carcinoma based on ultra-high performance liquid chromatography coupled with quadrupole time-of-flight mass spectrometry. Journal of Separation Science, 2014, 37:1728-1735.]. Principal component analysis (PCA) and orthogonal projection on latent structure discriminant analysis (OPLS-DA) were performed to visualize the metabolic alterations between lung cancer patients and healthy controls after mean centering and unit variance scaling. Pareto scaling was further used for OPLS-DA. The OPLS-DA model was adapted after performing a permutation test running 200 times, including the default seven-round cross-validation with one-seventh of the samples being excluded in each round to rule out overfitting. The variable importance in projection values (VIPs) and p values of Student's t-test were used to measure the significance of each variable. Metabolites with VIP > 1 and $p < 0.05$ were identified as different metabolites between two groups. MetaboAnalyst 4.0 was used to identify a variety of functional enrichment analysis and metabolic pathways.

B. Multivariate Modeling: Healthy Control vs. Stage I & II Lung Cancer, Healthy Control vs. Stage III & IV Lung Cancer

In order to establish a discriminant model for identifying early-stage lung cancer from healthy people, a discovery cohort of 50 healthy control samples and 35 early-stage (stage I and stage II) samples was adopted to find prominent metabolites and train the discriminant model. A held-out validation cohort consisting of 25 healthy controls and 17 early-stage cancer patients was used to validate the performance of the well-trained models. Table 4 describes the discriminative model involving the five best significant plasma metabolites identified from the above section: Palmitic acid, Heptadecanoic acid, Ornithine, Tridecanoic acid, and Stearic acid.

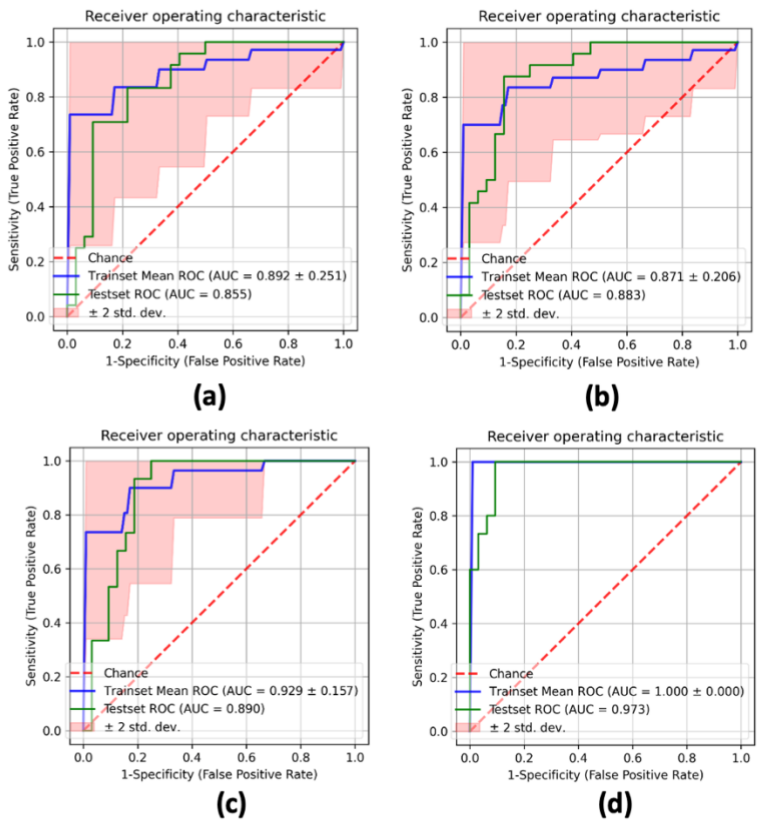

Figure S6 (a)-(b) Receiver-operating characteristic (ROC) curves for discriminating healthy controls and early-stage lung cancer patients **[(a)** ROC curves of the logistic model using top 5 metabolomic markers; **(b)** ROC curves of the logistic model using top 10 metabolomic markers]. **(c)-(d)** Receiver-operating characteristic (ROC) curves for discriminating healthy controls and advanced-stage lung cancer patients **[(c)** ROC curves of the logistic model using top 5 metabolomic markers; **(d)** ROC curves of the logistic model using top 10 metabolomic markers].

Table S6. Performance of logistic regression models with various biomarkers for discriminating healthy controls and early-stage lung cancer patients.

	**Classifier with the top five significant metabolites**		**Classifier with the top ten significant metabolites**	
	Discovery	Validation	Discovery	Validation
**AUC**	0.892 (+/- 0.251)	0.855	0.871 (+/- 0.206)	0.883
**Accuracy**	0.805 (+/- 0.240)	0.768	0.808 (+/- 0.236)	0.857
**Precision**	0.843 (+/- 0.398)	0.769	0.852 (+/- 0.377)	0.860

**Recall**	0.736 (+/- 0.291)	0.768	0.736 (+/- 0.395)	0.857

Using the metabolites in Table S6, a linear relationship, log(P/(1-P)) = 4.84E-05 − 3.62E-05 × Palmitic acid − 7.29E-04 × Heptadecanoic acid + 6.43E-03 × Ornithine− 1.92E-02 × Tridecanoic acid + 1.48E-05 × Stearic acid, was used for calculations. The optimal threshold (cut-off point) for the above logistic model is 0.338. From Figures S6 (a) (b) and Table S6, we can see that after using this held-out validation, the AUC can increase from 85.5% to 88.7% using top ten significant metabolites to identifying early-stage lung cancer from healthy people.

We also established a discriminant model for identifying advanced-stage lung cancer from healthy people. A discovery cohort of 50 healthy control samples and 29 advanced-stage (stage III and stage IV) samples was adopted to find prominent metabolites and train the discriminant model. A held-out validation cohort consisting of 25 healthy controls and 15 advanced-stage cancer patients was used to validate the performance of the well-trained model.

Table S7. Performance of logistic regression models with various biomarkers for discriminating healthy controls and advanced-stage lung cancer patients.

	**Classifier with the top five significant metabolites**		**Classifier with the top ten significant metabolites**	
	Discovery	Validation	Discovery	Validation
**AUC**	0.929 (+/- 0.157)	0.890	1.000 (+/- 0.000)	0.973
**Accuracy**	0.786 (+/- 0.245)	0.851	0.973 (+/- 0.086)	0.915
**Precision**	0.798 (+/- 0.376)	0.879	0.976 (+/- 0.117)	0.921
**Recall**	0.714 (+/- 0.306)	0.851	0.964 (+/- 0.175)	0.915

Using the metabolites in Table S7, a linear relationship, log(P/(1-P)) = 3.31E-05 − 4.22E-05 × Palmitic acid − 2.42E-03 × Heptadecanoic acid + 7.69E-03 × Ornithine− 9.44E-03 × Tridecanoic acid – 2.01E-07 × Stearic acid, was used. The optimal threshold (cut-off point) for the above logistic model is 0.271. The results from Figures S6 (c) (d) and Table S7, show that we can obtain a good model to discriminating healthy controls versus advanced-stage lung cancer patients, and healthy controls versus early-stage lung cancer patients separately with validation accuracy all above 0.85.

C. Linear Relationship for the Logistic Regression Models

- The linear relationship for discriminating lung cancer from healthy controls is:

log(P/(1-P)) = 3.07E-05 − 5.79E-05×Palmitic acid − 2.32E-03×Heptadecanoic acid + 5.91E-03×4-Oxoproline −1.17E-02×Tridecanoic acid + 6.19E-03×Ornithine.

The optimal threshold (cut-off point) for the above logistic regression model is 0.572.

- The linear relationship for the health stage is

log(P/(1-P)) = –1.84E-06 + 2.01E-05 ×Palmitic acid + 6.83E-04 ×Heptadecanoic acid – 2.91E-03 ×Ornithine + 1.50E-04 ×Tridecanoic acid + 2.38E-05 ×Stearic acid.

The optimal threshold (cut-off point) for the above model is 0.541.

- The linear relationship for the early-stage is

log(P/(1-P)) = 1.25E-06 – 1.01E-06 ×Palmitic acid – 1.34E-04 ×Heptadecanoic acid + 1.21E-03 ×Ornithine – 1.03E-04 ×Tridecanoic acid –2.57E-05 ×Stearic acid.

The optimal threshold (cut-off point) for the above model is 0.327.

- The linear relationship for the advanced-stage is

log(P/(1-P)) = 5.88E-07 – 1.91E-05 ×Palmitic acid –5.49E-04 ×Heptadecanoic acid + 1.70E-03 ×Ornithine – 4.68E-05 ×Tridecanoic acid + 1.95E-06 ×Stearic acid.

The optimal threshold (cut-off point) for the above model is 0.238.
